# CRH^CeA→VTA^ inputs inhibit the positive ensembles to induce negative effect of opiate withdrawal

**DOI:** 10.1038/s41380-021-01321-9

**Published:** 2021-10-12

**Authors:** Changyou Jiang, Xiao Yang, Guanhong He, Fan Wang, Zhilin Wang, Wendong Xu, Ying Mao, Lan Ma, Feifei Wang

**Affiliations:** Department of Neurosurgery, Huashan Hospital, State Key Laboratory of Medical Neurobiology and MOE Frontiers Center for Brain Science, School of Basic Medical Sciences, Institutes of Brain Science, Fudan University, Shanghai, 200032 China

**Keywords:** Addiction, Neuroscience

## Abstract

Plasticity of neurons in the ventral tegmental area (VTA) is critical for establishment of drug dependence. However, the remodeling of the circuits mediating the transition between positive and negative effect remains unclear. Here, we used neuronal activity-dependent labeling technique to characterize and temporarily control the VTA neuronal ensembles recruited by the initial morphine exposure (morphine-positive ensembles, Mor-Ens). Mor-Ens preferentially projected to NAc, and induced dopamine-dependent positive reinforcement. Electrophysiology and rabies viral tracing revealed the preferential connections between the VTA-projective corticotrophin-releasing hormone (CRH) neurons of central amygdala (CRH^CeA→VTA^) and Mor-Ens, which was enhanced after escalating morphine exposure and mediated the negative effect during opiate withdrawal. Pharmacologic intervention or CRISPR-mediated repression of CRHR1 in Mor-Ens weakened the inhibitory CRH^CeA→VTA^ inputs, and alleviated the negative effect during opiate withdrawal. These data suggest that neurons encoding opioid reward experience are inhibited by enhanced CRH^CeA→VTA^ inputs induced by chronic morphine exposure, leading to negative effect during opiate withdrawal, and provide new insight into the pathological changes in VTA plasticity after drug abuse and mechanism of opiate dependence.

## Introduction

Drugs of abuse (e.g., cocaine, opiates) trigger the initial acute-reward effect, lead to adaptive changes in the brain function following administration [1–3], and produce unpleasant physical and negative effects, including dysphoria, depression, irritability, and anxiety after the termination of use [4, 5]. Both the reward effect of the drug and the desire to avoid the unpleasant somatic symptoms or emotional feelings drive alternating rounds of positive and negative reinforcement for maintaining drug dependence, respectively [6]. For example, the clinical use of morphine, the most potent analgesic for chronic pain, has been limited due to severe withdrawal symptoms and a high risk of relapse [6, 7]. The transition to a drug-dependent state is accompanied by a series of changes in the plasticity of brain circuits [8], which triggers the pathological changes in the emotional processing.

Neurons in the ventral tegmental area (VTA) can be activated by emotional stimuli (i.e., positive/negative valence, high/low arousal) such as reward and aversion, and mediate the expression of the adaptively appropriate behavior [9–11]. Recent studies showed that synaptic inputs to the VTA from the laterodorsal tegmentum and the lateral habenula drive reward and aversion in mice, respectively [12, 13]. The establishment of an opiate-dependent state and aversive withdrawal symptoms is dependent on the plasticity of VTA [14, 15]. The diversity of GABAergic inputs modulates the activity of VTA dopaminergic neurons [16–19]. Dopaminergic neurons are activated by disinhibition of GABAergic projections from the RMTg during morphine withdrawal [20–22]. The transition from an opiate-naive to an opiate-dependent state is associated with a change, from an inhibitory to an excitatory response, of the GABA_A_ receptors on GABAergic neurons in the VTA [23, 24]. These studies indicate that heterogeneous inhibitory inputs to the VTA participate in the development of opioid dependence.

Drug dependence has been hypothesized to be driven by two relatively independent systems: the downregulation of the dopamine (DA)-reward system (DA function) in the VTA—a within-system neuronal adaptation that leads to anhedonia, and the upregulation of the stress system—a between-system neuronal adaptation that leads to stress disorders. Corticotrophin-releasing hormone (CRH) system has been shown to be involved in neuroplasticity changes evoked during drug withdrawal [25, 26]; however, how CRH neurons participate in the synaptic inhibition of the reward system following chronic opiate exposure remains undetermined.

In this study, we specifically labeled neuronal ensembles recruited by initial morphine exposure in the VTA, taking the advantage of the immediate early gene-based, synaptic and neuronal activity-responsive systems. Our data reveal that chronic morphine administration preferentially enhanced the GABAergic inputs of CRH neurons in the central amygdala (CeA) to VTA dopaminergic morphine ensembles, which was essential for the development of the negative effect during opiate withdrawal. Our results highlight the importance of CRH neuron-mediated functional connectivity between CeA and VTA, which reduces morphine euphoria and drives the negative effect via enhanced GABAergic transmission onto the VTA ensembles encoding a drug-reward experience.

## Results

### The activation of VTA neuronal ensembles recruited by morphine induces DA releasing in NAc and positive reinforcement

**Fig. 1:**
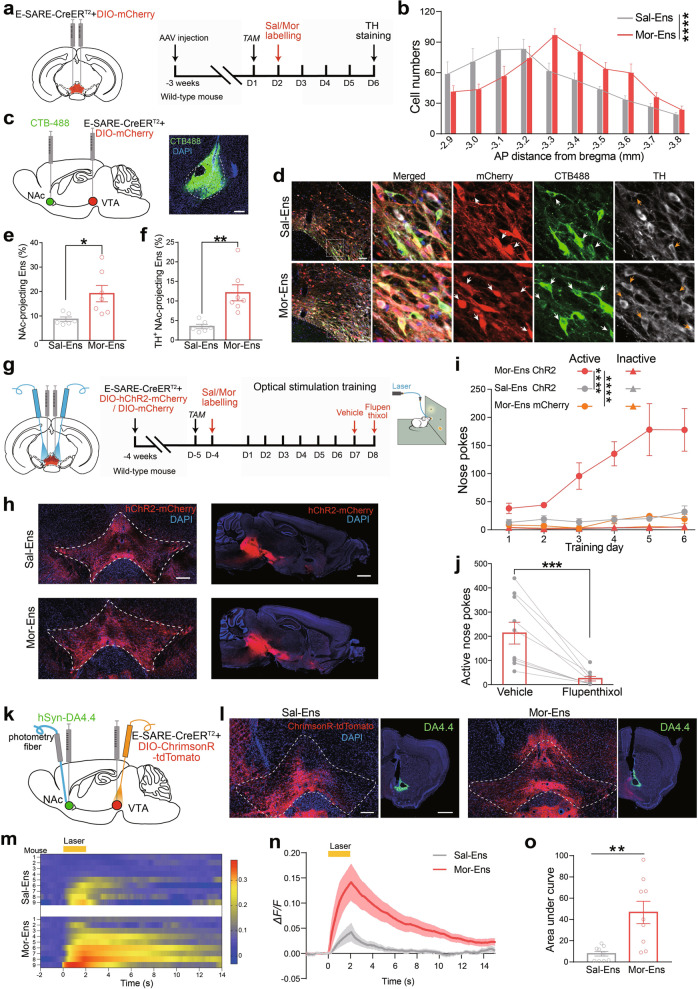
Mor-Ens drive positive reinforcement in a DA-dependent manner. **a** Experimental process of ensemble labeling. Mice were intraperitoneally injected with TAM (125 mg/kg), and morphine (10 mg/kg) or saline was injected 24 h later. Immunostaining was carried out four days after the labeling. **b** Quantification of the number of Mor-Ens and Sal-Ens in the VTA across the anterior–posterior axis (−2.9 to −3.8 mm from bregma) (*n* = 7 mice per group). Two-way RM ANOVA, F_(9, 108)_ = 6.880, *P* < 0.0001. **c** Schematic of virus injection and representative images of CTB-488 injection in NAc. Scale bar, 200 μm. **d** Representative images of TH staining in the slice containing VTA. Red: mCherry; Green: CTB-488; Gray: TH; Blue: DAPI. Scale bar, 50 μm, 10 μm. White arrowheads indicated the NAc-projecting ensembles, orange arrowheads indicated the TH^+^ NAc-projecting ensembles. **e** Percentage of NAc-projecting cells in labeled VTA ensembles. Unpaired *t* test, *t* = 2.805, *df* = 11, *P* = 0.0171. **f** Percentage of TH^+^ cells in labeled NAc-projecting ensembles. Mann–Whitney U test, *U* = 3.5, *P* = 0.0099. **g** Schematic of ICSS task. Mice were trained to ICSS with 473 nm laser (20 Hz, 5 ms, 2-s duration) by nose pokes four days after the ensemble labeling. Vehicle and flupenthixol (0.5 mg/kg) were intraperitoneally administrated at the 7th and 8th training sessions. **h** Representative images of the coronal and sagittal slices showing the restrict expression of ChR2-mCherry in the VTA and their projecting terminals in the NAc. Red: mCherry; Blue: DAPI. Scale bar, left: 200 μm; right: 1000 μm. **i** Numbers of active and inactive nose pokes in ICSS training. Two-way RM ANOVA, F_(5, 80)_ = 7.197, *P* < 0.0001 for Sal-Ens ChR2 vs Mor-Ens ChR2; F_(5, 80)_ = 6.725, *P* < 0.0001 for Mor-Ens ChR2 vs Mor-Ens mCherry. **j** Average active nose pokes in a 60-min test session on day 7 and day 8 (pharmacological intervention). Paired *t* test, *t* = 4.821, *df* = 9, *P* = 0.0009. **k** Schematic of virus injection and fiber photometry recordings of DA4.4. **l** Representative coronal images showing the expression of ChrimsonR–tdTomato in the VTA, and expression of DA4.4 in the NAc. Green: DA4.4; Red: tdTomato; Blue: DAPI. Scale bars, left: 200 μm, right: 500 μm. **m** Heatmap of DA 4.4 fluorescence in the NAc in response to Sal-Ens and Mor-Ens activation (594 nm, 20 Hz, 5-ms pulse width, 2-s duration, 20-s interval, 15 trails). **n**, **o** Average *ΔF*/*F* of DA4.4 fluorescence in response to optical stimulation of Sal-Ens or Mor-Ens (**n**) and the area under the curve (**o**). *n* = 9 mice per group. Unpaired *t* test, *t* = 3.631, *df* = 16, *P* = 0.0022. *P < 0.05, **P < 0.01, ***P < 0.001, ****P < 0.0001. Data are presented as mean ± SEM.

We used adeno-associated virus (AAV) expressing Cre^ERT2^ driven by enhanced synaptic activity-responsive element (*AAV-E-SARE-Cre*^*ERT2*^) and *AAV-DIO-mCherry* to label VTA neuronal ensembles activated by saline or morphine exposure. Mice were given a single intraperitoneal injection (i.p.) of saline or 10 mg/kg morphine at various times after tamoxifen (TAM) induction in the home cage. Ensembles were efficiently labeled 24–36 h after TAM induction (Supplementary Fig. 1a–d). The specific recruitment of the Sal-Ens and Mor-Ens by ESARE-triggered approach was verified by c-Fos staining following reexposure to saline or morphine, respectively (Supplementary Fig. 1e, f). Quantification of the ensembles labeled by saline (Sal-Ens) and morphine (Mor-Ens) across the anterior–posterior axis of the VTA (Fig. 1a, b and Supplementary Fig. 2a–d) showed that Sal-Ens was enriched in rostral VTA, and Mor-Ens was relatively enriched in medium VTA (Fig. 1b). Both Mor-Ens and Sal-Ens sent the majority of collaterals to the nucleus accumbens (NAc), lateral habenula (LHb), amygdala, and medial prefrontal cortex (mPFC). The normalized terminal intensity of the ensembles indicated that Mor-Ens terminals were more abundant in the NAc, while they showed no difference in the LHb, mPFC and amygdala compared with that of the Sal-Ens (Supplementary Fig. 2e, f). To validate and quantify the NAc-projecting ensembles in the VTA, the green retrograde tracer (cholera toxin B subunit, CTB-488) was injected into the NAc of mice, and the tagged ensembles in the VTA were analyzed (Fig. 1c, d). The percentage of retrogradely labeled cells in Mor-Ens was higher than that in Sal-Ens (Fig. 1e). Triple labeling with tyrosine hydroxylase (TH) staining indicated that the percentage of dopaminergic NAc-projecting Mor-Ens was greater than that in Sal-Ens (Fig. 1f). These results show that VTA Mor-Ens form more dopaminergic connections to NAc, as compared with Sal-Ens.

Instrumental reinforcement procedures combined with chemogenetic activation of the ensembles were used to evaluate the reinforcement effect of the morphine ensembles. Mice infected with *AAV-E-SARE-Cre*^*ERT2*^ and *AAV-DIO-hM3Dq-mCherry* or *AAV-DIO-mCherry* in the VTA were conditioned in a place-preference chamber after clozapine N-oxide (CNO) injection to specifically activate VTA Sal- or Mor-Ens in vivo (Supplementary Fig. 3a, b). Mice in Mor-Ens group showed significant preference for the CNO-paired chamber, more time spent in open arm, without affecting locomotor activity and saccharin preference, compared with the Sal-Ens group (Supplementary Fig. 3c–f).

Virus infected mice implanted with an optic fiber above the VTA were trained to intracranial self-stimulation (ICSS) with 473 nm laser (20 Hz, 5 ms) by nose pokes after ensemble labeling (Fig. 1g, h). The number of nose pokes coupled to optical stimulation in mice of Mor-Ens group increased during training trials, and reached to a high level at training days 5–6, in contrast to unchanged nose pokes throughout training trials in Sal-Ens group or control mice lacking ChR2 (Fig. 1i). Pretreatment with DA-receptor antagonist flupenthixol (0.5 mg/kg, i.p.) [27] significantly attenuated nose pokes associated with laser stimulation of Mor-Ens (Fig. 1j). Dopamine (DA) release was determined by recording fluorescence dynamics of DA4.4, a DA sensor [28, 29] (Fig. 1k, l). We observed a concordant increase of DA sensor fluorescence in the NAc when activating Mor-Ens, while activating Sal-Ens induced DA release at a much lower level (Fig. 1m–o). These data show that DA release in NAc from terminals of the activated Mor-Ens is crucial for positive reinforcement.

### Chronic morphine enhances inhibitory synaptic transmission onto Mor-Ens, and this is involved in the development of the negative effect during opiate withdrawal

**Fig. 2:**
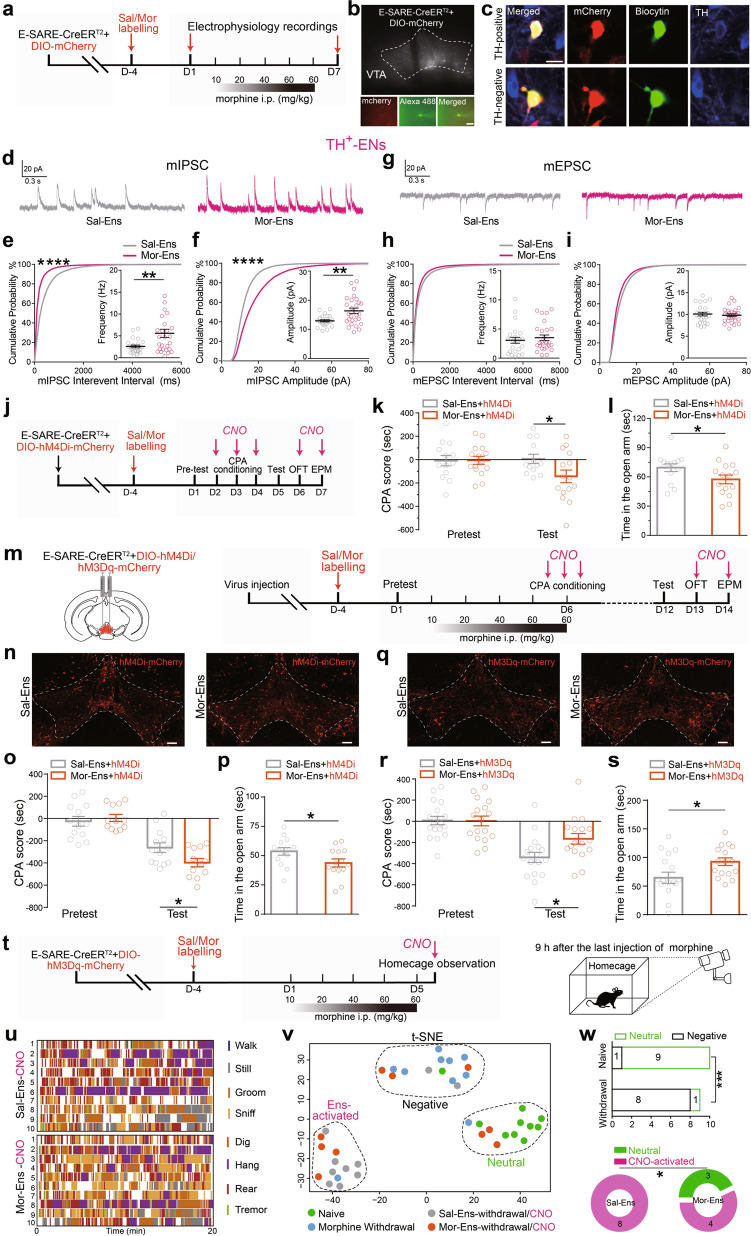
The inhibitory transmission in dopaminergic Mor-Ens is enhanced and this is required for the development of negative effect during opiate withdrawal. **a** Experimental scheme of the VTA ensembles recording before (day 1), or after the escalating dose of morphine treatment (day 7). **b** Representative images of neurons for the recordings in the VTA and the neuron filled with dye. Scale bar, 20 μm. **c** Mor-Ens or Sal-Ens were injected with biocytin, and costained with anti-TH antibody after recording. Red: mCherry; Green: biocytin; Blue: TH. Scale bar, 10 μm. **d** Representative traces of mIPSCs. **e, f** Cumulative probability distribution and average amplitude and frequency of mIPSCs recorded from TH^+^ VTA ensembles after escalating-dose of morphine administration. **g** Representative traces of mEPSCs. **h, i** Cumulative probability distribution and average amplitude and frequency of mEPSCs recorded from TH^+^ VTA ensembles after escalating dose of morphine administration. Sal-Ens: 9 mice, Mor-Ens: 11 mice. Unpaired *t* test, frequency: *t* = 2.864, *df* = 49, *P* = 0.0061 in (**e**), amplitude: *t* = 3.237, *df* = 49, *P* = 0.0022 in (**f**); Mann–Whitney U test, frequency: *U* = 291, *P* = 0.5429 in (**h**), amplitude: *U* = 311, *P* = 0.8151 in (**i**); two-sample Kolmogorov–Smirnov (KS) test for cumulative probability distribution, interval: *P* < 0.0001 in (**e**), amplitude: *P* < 0.0001 in (**f**). **j–l** Schematic representation of the virus injection and behavioral tests in mice without withdrawal. The effect of inhibition of Mor-Ens in the VTA on CPA and anxiety (**j**). Quantification of CPA score in hM4Di groups (**k**). Two-way RM ANOVA, F_groups × session_ (1, 26) = 5.342, *P* = 0.0290, Sal-Ens vs Mor-Ens within test, *P* = 0.0397. (**l**) Quantification of the time in open arm in hM4Di groups without withdrawal. Mann–Whitney U test, *U* = 58, *P* = 0.0411. **m** Experimental process of the virus injection and behavioral tests after the ensemble labeling in withdrawal mice. **n–p** The effect of inhibition of Mor-Ens on morphine-withdrawal-induced CPA and anxiety. Representative images of expression of hM4Di-mCherry in VTA Sal-Ens and Mor-Ens (**n**). Scale bar, 100 μm. Quantification of morphine-withdrawal-induced CPA score in hM4Di groups (**o**). Two-way RM ANOVA, F_groups × session_ (1, 23) = 7.248, *P* = 0.013, Sal-Ens vs Mor-Ens within test, *P* = 0.0429. Quantification of the time in open arm in hM4Di groups (**p**). Mann–Whitney U test, *U* = 52, *P* = 0.0350. **q–s** The effect of activation of Mor-Ens on morphine-withdrawal-induced CPA and anxiety. Representative images of expression of hM3Dq-mCherry in VTA Sal-Ens and Mor-Ens (**q**). Scale bar, 100 μm. Quantification of morphine-withdrawal-induced CPA score in hM3Dq groups (**r**). Two-way RM ANOVA, F_groups × session_ (1, 33) = 5.125, *P* = 0.0303, Sal-Ens vs Mor-Ens within test, *P* = 0.0170. Quantification of the time in open arm in hM3Dq groups (**s**). Mann–Whitney U test, *U* = 73, *P* = 0.0387. **t** Experimental process of virus injection and 20-min behavioral recording in the home cages. **u** Analysis of the eight behaviors in mice that activation of Sal-Ens and Mor-Ens during the spontaneous withdrawal period. Each column represents individual behavior. Each row represents one mouse. **v** *t*-Distributed stochastic neighbor embedding (*t*-SNE) representation of 40 mice behavioral patterns showing three clusters: negative (black), neutral (green), and chemo-activated (magenta). **w** Top: Proportion of mice displaying neutral or negative pattern. Bottom: Proportion of CNO-treated mice displaying neutral pattern. Top, *χ*^2^ test, *P* = 0.0006; Bottom, *χ*^2^ test, *P* = 0.0384. *P < 0.05, **P < 0.01, ***P < 0.001, ****P < 0.0001. Data are presented as mean ± SEM.

Chronic opiate administration causes pathological changes of VTA plasticity. Therefore, whole-cell patch-clamp recordings were performed to evaluate synaptic remodeling of the ensembles after chronic morphine exposure (Fig. 2a–c). The mIPSC and mEPSC of Sal-Ens and Mor-Ens were not different before chronic morphine treatment (day 1) (Supplementary Fig. 4a–d). After the escalating dose of morphine administration (day 7), the mIPSC amplitude and frequency were increased significantly in TH^+^ population of Mor-Ens, compared with that in Sal-Ens (Fig. 2d–f), while no differences in mEPSC were observed (Fig. 2g–i). In TH^−^ population, mIPSC amplitude exhibited a minor decrease in Mor-Ens, compared with that in Sal-Ens (Supplementary Fig. 4e–j). These data indicate that the dopaminergic Mor-Ens receives more inhibitory inputs than Sal-Ens after chronic morphine exposure.

To assess whether the activity of Mor-Ens is critical for the negative effects, hM4Di was expressed in labeled Mor-Ens and Sal-Ens. In unlabeled mice, CNO treatment did not affect the development of conditioned place aversion (CPA), locomotor activity, the time spent in the open arm, and saccharin preference following morphine withdrawal (Supplementary Fig. 5a–e). In the groups with or without escalating dose of morphine treatment, inhibition of VTA Mor-Ens by CNO significantly increased the CPA score, and decreased the time spent in the open arms of the elevated-plus maze (EPM) (Figs. 2j–l, m–p), while having no effects on locomotor activity and saccharin preference (Supplementary Fig. 6d, e). Instead, when expressing hM3Dq in labeled Mor-Ens or Sal-Ens, activation of Mor-Ens by CNO during the place-aversion conditioning reduced the CPA score, increased the time spent in the open arms in EPM (Fig. 2q–s), while having no effects on locomotor activity and saccharin preference (Supplementary Fig. 6a–c). These results suggest that the activity of VTA Mor-Ens is required for the formation of conditioned aversion and anxiety.

Spontaneous morphine withdrawal evokes negative somatic symptoms. Behavioral patterns were assessed 9 hrs after the last injection of escalating dose of morphine (Fig. 2t). There were significant differences in walking, sniffing, rearing, and tremoring between morphine and saline treatment groups (Supplementary Fig. 6f, g). Analysis of the individual behaviors showed mild differences between activation of Mor- and Sal-Ens by CNO during spontaneous withdrawal (Fig. 2u and Supplementary Fig. 6h). However, evaluation of the behavior spectrum using *t*-distributed stochastic neighbor embedding (*t*-SNE) revealed three separated clusters: a negative cluster (consisting mostly of opiate-withdrawal individuals), a neutral cluster (consisting mostly of naive individuals, which did not undergo withdrawal and CNO activation), and a chemo-activated cluster, which was composed of individuals in which Sal- or Mor-Ens were activated by CNO during spontaneous withdrawal (Fig. 2v). Activation of Mor-Ens in mice withdrawal from morphine increased the percentage of individuals clustered into the neutral cluster (Fig. 2w). Taken together, these results suggest that activation of the ensembles recruited by the initial morphine exposure reduces conditioned aversion, alleviates negative somatic symptoms, and anxiety during opiate withdrawal.

### The activity of CRH^CeA→VTA^ neurons is increased following chronic morphine administration

**Fig. 3:**
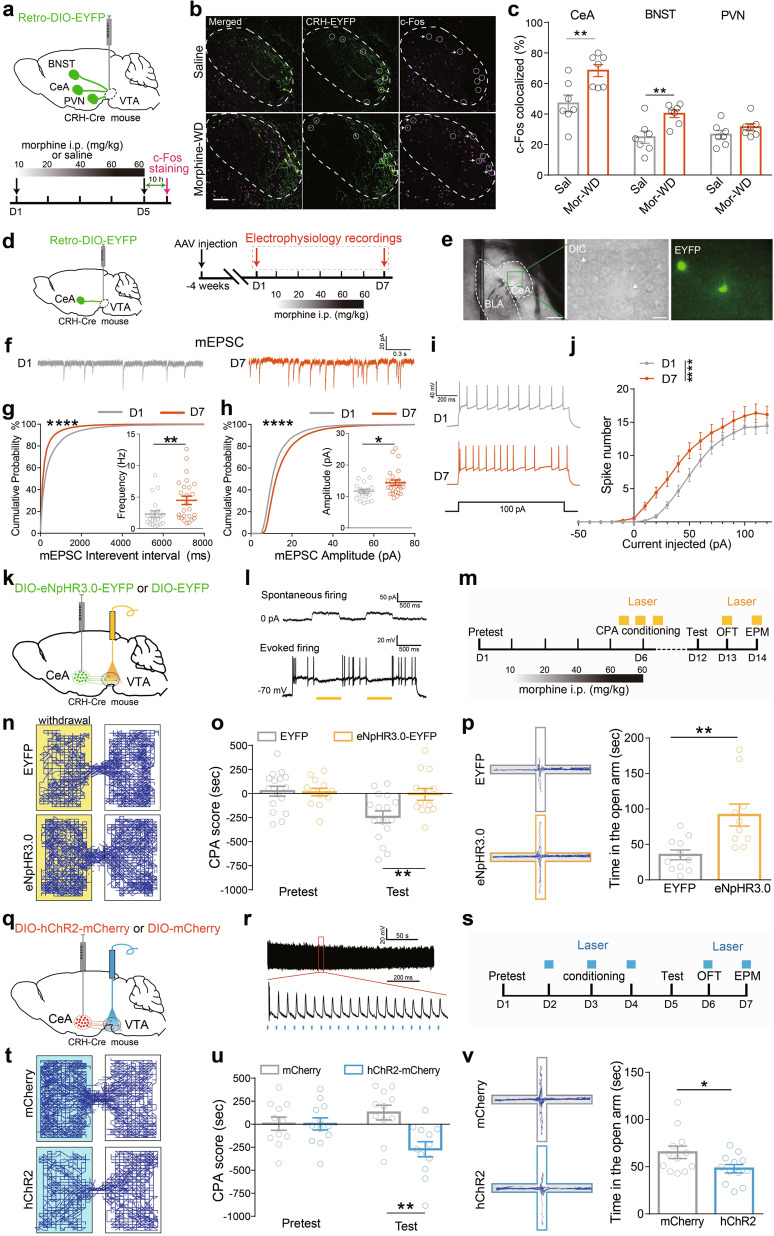
The activation of CRH^CeA→VTA^ neurons facilitates conditioned-place aversion and the anxiety during opiate withdrawal. **a** Experimental process of the virus injection and c-Fos staining in *CRH-ires-Cre* mouse. **b, c** Representative images of the CeA (**b**) and quantification of the c-Fos colocalization in EYFP^+^ cells of the indicated brain regions (**c**). Green: EYFP; Magenta: c-Fos. Scale bar, 100 μm. Unpaired *t* test, CeA: *t* = 3.219, *df* = 12, *P* = 0.0074; BNST: *t* = 3.283, *df* = 12, *P* = 0.0065; PVN: *t* = 1.33, *df* = 12, *P* = 0.2083. **d** Experimental scheme depicts the virus injection and the recording of CRH^CeA→VTA^ neurons in *CRH-ires-Cre* mouse. **e** Representative images of EYFP^+^ neurons in the CeA. White dashes labeled the CeA and BLA structure. Scale bar, 200 μm. **f–h** Representative traces (**f**) and the cumulative probability distribution or average amplitude and frequency of mEPSCs (**g, h**) recorded from CRH^CeA→VTA^ neurons before (D1) and after escalating dose of morphine administration (D7). Mann–Whitney *U* test, frequency: *U* = 136, *P* = 0.0086 in (**g**), amplitude: *U* = 145, *P* = 0.0159 in (**h**); two-sample KS test for interval: *P* < 0.0001, amplitude: *P* < 0.0001. **i, j** Representative AP traces (**i**) and quantification of the induced spike number (**j**) of the CRH^CeA→VTA^ neurons before (D1) and after escalating dose of morphine administration (D7). In total, 23–27 neurons from 6 mice per group. Two-way ANOVA, *Bonferroni’s* post hoc test, F_(2, 1364)_ = 37.58, *P* < 0.0001. **k** Schematic representation of virus injection and optic-fiber implantation of *CRH-ires-Cre* mice. **l** Spontaneous and evoked firings of eNpHR3.0^+^ CRH neurons are interrupted by the pulses of 594-nm laser. **m** Experimental process of the optical inhibition during the behavioral assays. **n–p** The effect of optical inhibition of CRH^CeA→VTA^ terminals during morphine-withdrawal-induced CPA (**o**) and anxiety (**p**). Representative tracks illustrate the CPA test sections (**n**). Two-way RM ANOVA, F_virus × session_ (1, 27) = 4.553, *P* = 0.0430, EYFP vs eNpHR3.0 within test, *P* < 0.01 in (**o**); unpaired *t* test, *t* = 3.413, *df* = 19, *P* = 0.0029 in (**p**). **q** Schematic representation of the virus injection and optic-fiber implantation in *CRH-ires-Cre* mice. **r** The action potentials of a CRH neuron in the CeA induced by 473-nm laser (20 Hz, 5 ms). **s** Experimental process of the optical activation during the behavioral assays. **t–v** Optical stimulation of the CRH^CeA→VTA^ terminals drives CPA (**u**) and anxiety-like behavior (**v**). Representative tracks (**t**) illustrate the CPA test section. Two-way RM ANOVA, F_virus × session_ (1, 21) = 7.677, *P* = 0.0115, mCherry vs hChR2 within test, *P* < 0.01 in (**u**); unpaired *t* test, *t* = 2.195, *df* = 22, *P* = 0.0390 in (**v**). *P < 0.05, **P < 0. 01, ****P < 0.0001. Data are presented as mean ± SEM.

Upregulation of the brain-stress system and downregulation of the reward system have been found, and corticotrophin-releasing hormone (CRH) has been implicated in behavioral responses during acute and chronic withdrawal [26, 30, 31]. *AAV-Retro-EF1a-DIO-EYFP* was infected into the VTA of *CRH-ires-Cre* mice, and VTA-projecting CRH neurons tagged with EYFP in the CeA, the bed nucleus of stria terminals (BNST), and the paraventricular nucleus of the hypothalamus (PVN) were detected. c-Fos expression was increased in CRH^CeA→VTA^ and CRH^BNST→VTA^ neurons, while unchanged in CRH^PVN→VTA^ neurons after chronic morphine treatment (Fig. 3a–c), indicating that CRH^CeA→VTA^ and CRH^BNST→VTA^ neurons are activated during opiate withdrawal.

CeA contains primarily inhibitory outputs and orchestrates adaptive responses to emotional events [32–34]. To assess whether the inhibitory CRH^CeA→VTA^ inputs changes following chronic morphine administration, *CRH-ires-Cre* mice were infected with *AAV-Retro-EF1a-DIO-EYFP* in the VTA to label CRH^CeA→VTA^ CRH neurons. mEPSC, mIPSC and evoked action potential (AP) in CRH^CeA→VTA^ neurons were recorded (Fig. 3d, e). The frequency and amplitude of mEPSC in CRH^CeA→VTA^ neurons were increased following escalating dose of morphine administration, while mIPSC was not changed (Fig. 3f–h and Supplementary Fig. 7a–c). Additionally, chronic morphine treatment increased excitability (Fig. 3i, j), reduced AP threshold, and increased input resistance of the CRH^CeA→VTA^ neurons (Supplementary Fig. 7d, e), but had no effect on after-hyperpolarization potential, amplitude, and half width of APs in these neurons (Supplementary Fig. 7f–h). These results suggest that the activation, excitatory transmission and membrane excitability of CRH^CeA→VTA^ neurons are enhanced following chronic morphine administration.

### The activation of CRH^CeA→VTA^ terminals increases anxiety and drives conditioned-place aversion induced by opiate withdrawal

The effect of activation of CRH^CeA→VTA^ projections on morphine-withdrawal-induced CPA and anxiety was then examined. *CRH-ires-Cre* mice were infected with *AAV-DIO-eNpHR3.0-EYFP* in the CeA (Fig. 3k, l and Supplementary Fig. 8a, b). Optogenetic inhibition of CRH^CeA→VTA^ terminals by 594-nm laser stimulation during conditioning decreased conditioned aversion induced by opiate withdrawal (Fig. 3m–o). Optogenetic inhibition of CRH^CeA→VTA^ terminals increased the time spent in the open arm (Fig. 3p), while it did not affect the locomotor activity (Supplementary Fig. 8d). The above data suggest that the activation of CRH^CeA→VTA^ terminals promotes anxiety level and the formation of conditioned aversion induced by opiate withdrawal.

To examine the role of CRH^CeA→VTA^ projections in the development of negative effect, *CRH-ires-Cre* mice were bilaterally infected with *AAV-DIO-hChR2-mCherry* in the CeA and implanted optic fibers above the VTA to activate CRH ^CeA→VTA^ terminals (Fig. 3q, r and Supplementary Fig. 8c). Optical activation of CRH^CeA→VTA^ terminals by 473-nm laser induced a place aversion (Fig. 3s–u) and decreased the time spent in the open arms in the EPM test (Fig. 3v), while it had no effect on locomotor activity (Supplementary Fig. 8e). These experiments indicate that the increased activity of CRH^CeA→VTA^-projective neurons contributes to the development of CPA and anxiety during opiate withdrawal.

### Specific remodeling of the circuits of CRH^CeA→VTA^ neurons and Mor-Ens by chronic morphine administration

**Fig. 4:**
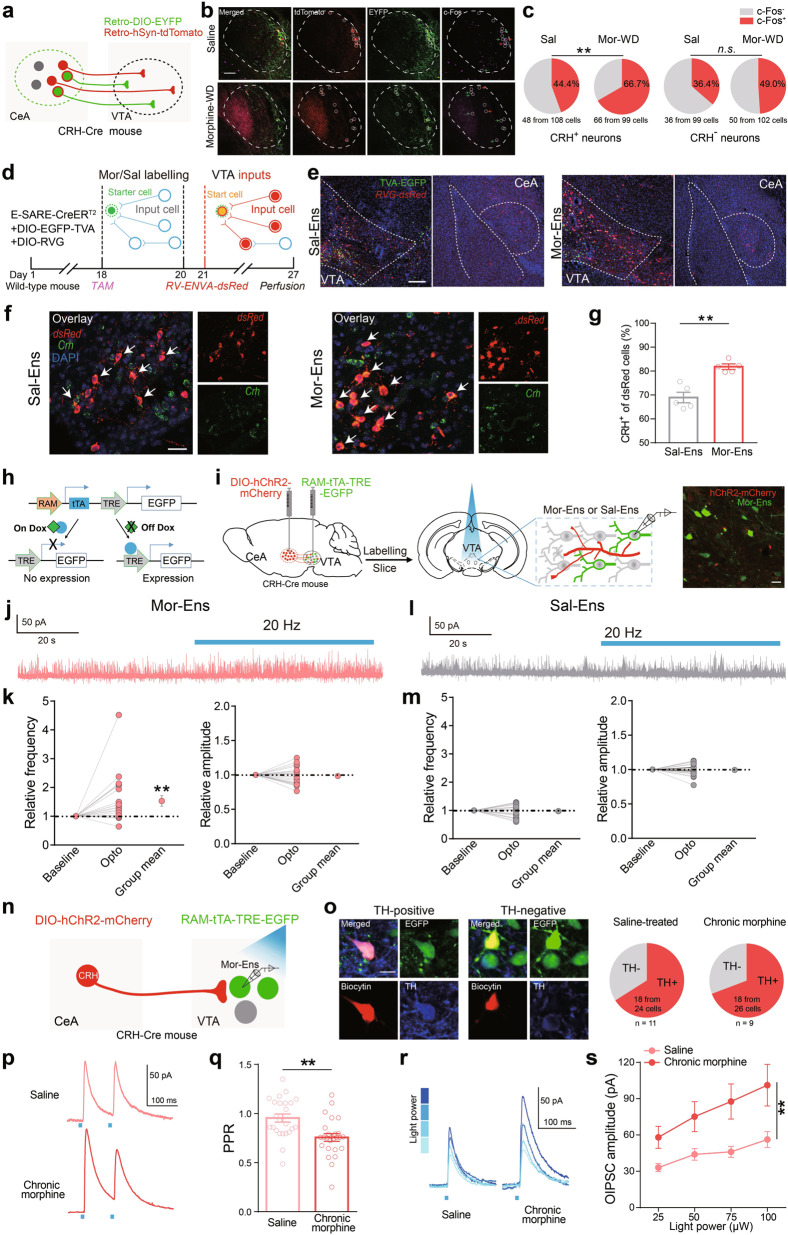
Specific remodeling of the circuits between CRH^CeA→VTA^ neurons and Mor-Ens by chronic morphine administration. **a** Schematic representation of the CeA→VTA-projecting neuron labeling. **b** Representative images of CRH^+^ (EYFP^+^), CRH^−^ (EYFP^-^ tdTomato^+^) neurons in the CeA colocalized with c-Fos. Green: EYFP; Red: tdTomato; Magenta: c-Fos. Scale bar: 100 μm. **c** Quantification of the percentage of c-Fos^+^ cells in CRH^+^ and CRH^−^ neurons after escalating dose of morphine administration. In total, 19–22 slices from 7 mice per group. Left, *χ*^2^ test, *P* = 0.0013; right, *χ*^2^ test, *P* = 0.0698. **d** Schematic representation of the rabies-mediated monosynaptic retrograde labeling of the inputs onto Sal-Ens or Mor-Ens in the VTA. **e** Representative images of Sal-Ens or Mor-Ens starters (EGFP^+^dsRed^+^ neurons) in the VTA, and input cells (red) in the CeA. Red: *dsRed*; Green: EGFP; Blue: DAPI. Scale bar, 100 μm. **f** Representative images of input cells in the CeA colocalized with *Crh* (green) mRNA probe, Red: *dsRed*; Green: *Crh*; Blue: DAPI. Scale bar, 20 μm. **g** The proportion of CRH^+^ neurons in CeA projecting to Sal-Ens and Mor-Ens. Sal-Ens: 209 CRH^+^ from 293 cells; Mor-Ens: 199 CRH^+^ from 244 cells, five mice per group. Mann-Whitney *U* test, *U* = 0, *P* = 0.008. **h** Schematic representation of *AAV-RAM-tTA-TRE-EGFP* vector. **i** Schematic representation of the IPSC recordings from the Mor-Ens or Sal-Ens. Right: the expression of CRH^hChR2-mCherry^ terminals and EGFP^+^ Mor-Ens in the VTA. Scale bar, 20 μm. **j, l** Representative traces of the currents in Mor-Ens or Sal-Ens neurons following 473-nm optical stimulation (20 Hz, 5 ms). **k, m** The relative changes of IPSC frequency and amplitude in Mor-Ens or Sal-Ens following optical stimulation. Paired *t* test, Mor-Ens: frequency, *t* = 2.923, *df* = 20, *P* = 0.0084; amplitude, *t* = 0.5531, *df* = 20, *P* = 0.5863. Sal-Ens: frequency, *t* = 0.4461, *df* = 19, *P* = 0.6606; amplitude, *t* = 0.3156, *df* = 19, *P* = 0.7558. **n** Schematic representation of optical stimulation and electrophysiological recordings. **o** Responsive Mor-Ens from slice of mice treated with saline or escalating dose of morphine was filled with biocytin and stained with anti-TH antibody after recording to identify the TH^+^ subtypes (Saline: *n* = 24 neurons from 11 mice; escalating dose of morphine: *n* = 26 neurons from 9 mice). Red: biocytin; Green: EGFP; Blue: TH. Scale bar, 10 μm. **p, q** Representative traces of PPR (**p**) and quantification of PPR values (**q**) in Mor-Ens response to optical stimulation (473 nm, 2-ms pulse width at 100-ms interval) on CRH^hChR2-mCherry^ terminals in the VTA. Unpaired *t* test, *t* = 3.554, *df* = 47, *P* = 0.001. **r, s** Representative traces (**r**) and quantification of amplitudes (**s**) of O-IPSCs in Mor-Ens in response to different laser intensities (Saline: *n* = 24 neurons from 11 mice; escalating dose of morphine: *n* = 22 neurons from 9 mice). Two-way ANOVA, F_(3, 176)_ = 5.112, *P* = 0.0020. **P < 0.001. Data are presented as mean ± SEM.

*CRH-ires-Cre* mouse was infected with *AAV-Retro-EF1a-DIO-EYFP* and *AAV-Retro-hSyn-tdTomato* in the VTA to label VTA-innervating neurons. c-Fos expression in CRH^+^ (EYFP^+^) or CRH^−^ (EYFP^−^ tdTomato^+^) neurons in the CeA was assessed (Fig. 4a, b). The proportion of c-Fos^+^ CRH^**+**^ neurons was increased after chronic morphine administration, while the proportion of c-Fos^+^ CRH^**-**^ neurons was not changed (Fig. 4c), indicating the specific regulation of CRH^CeA→VTA^ neuron activity by chronic morphine administration.

To investigate the monosynaptic inputs from the CeA to Sal-Ens or Mor-Ens, *C57 BL/6* mice were infected with *AAV-E-SARE-Cre*^*ERT2*^, and Cre-dependent helper viruses (*AAV-DIO-H2B-EGFP-TVA* and *AAV-DIO-RVG*) in the VTA. The rabies virus *RV-EnvA-dsRed* was injected one day after the ensembles labeling (Fig. 4d). The starter ensembles expressing EGFP and dsRed were restricted in the VTA (Fig. 4e) and costained with anti-TH antibody to identify the cell type composition (Supplementary Fig. 9a, b). The distribution of dsRed^+^ neurons showed that inputs cells were concentrated in the NAc, LHb, dorsal striatum and lateral hypothalamus (Supplementary Fig. 9c), which was consistent with previous reports [27, 35, 36]. The relative number of dsRed^+^ input neurons in the CeA was not different between Sal-Ens and Mor-Ens groups (Supplementary Fig. 9c). Fluorescent probes targeting *Crh* and *dsRed* mRNA were used to identify the CRH neurons in the CeA connecting to Sal- or Mor-Ens. The proportion of *crh*^+^ population in Mor-Ens connective CeA neurons was higher than that in Sal-Ens (Fig. 4f, g), suggesting that CRH^CeA→VTA^ inputs preferentially form synaptic connections with the Mor-Ens.

To achieve specific manipulation of CRH neurons and VTA ensembles in the same mouse, we used a doxycycline (Dox)-dependent *AAV****-****RAM-tTA-TRE-EGFP* system to label the ensembles (Fig. 4h). *CRH-ires-Cre* mice were infected with *AAV-DIO-hChR2-mCherry* in the CeA, and *RAM-tTA-TRE-EGFP* in the VTA (Fig. 4i). The RAM-captured ensembles (EGFP^+^ neurons) were largely overlapped with E-SARE-captured ensembles (mCherry^+^ neurons) recruited by saline or morphine, and showed similar spatial distribution and comparable cell number to those labeled by E-SARE system (Supplementary Fig. 10a–d). Instead, there were less than 30% overlapping distributions of the *E-SARE-*captured Mor-Ens with *RAM-*captured Sal-Ens in the same mouse (Supplementary Fig. 10e–g), indicating they were separated subpopulations. Synaptic responses of EGFP^+^ Sal-Ens and Mor-Ens, as well as the adjacent EGFP^−^ neurons that were not recruited by initial morphine exposure (Non-Mor-Ens) with optical stimulation were recorded (Fig. 4i). Activation of CRH^CeA→VTA^ terminals by 20-Hz optical stimulation increased the frequency of IPSCs in Mor-Ens (Fig. 4j, k), while it had no effect on that in Sal-Ens (Fig. 4l, m). Additionally, the amplitude of IPSC in Non-Mor-Ens was slightly decreased by activation of CRH^CeA→VTA^ terminals (Supplementary Fig. 10h, i). To investigate the effect of chronic morphine administration on the inhibitory CRH^CeA→VTA^ transmission in Mor-Ens, we recorded the O-IPSCs in Mor-Ens in response to the 473-nm laser, and identified the dopaminergic Mor-Ens via backfill staining (Fig. 4n, o). We found that majority of the responsive neurons were dopaminergic neurons, and Mor-Ens exhibited decreased paired-pulse ratio (PPR) value and increased O-IPSC amplitude after chronic morphine administration (Fig. 4p–s), revealing that the enhanced inhibitory tone from CRH^CeA→VTA^ projections to dopaminergic Mor-Ens following chronic morphine administration involves both presynaptic and postsynaptic mechanisms.

### Specific modulation of CRH^CeA→VTA^ and Mor-Ens pathway is required for the development of negative effect

**Fig. 5:**
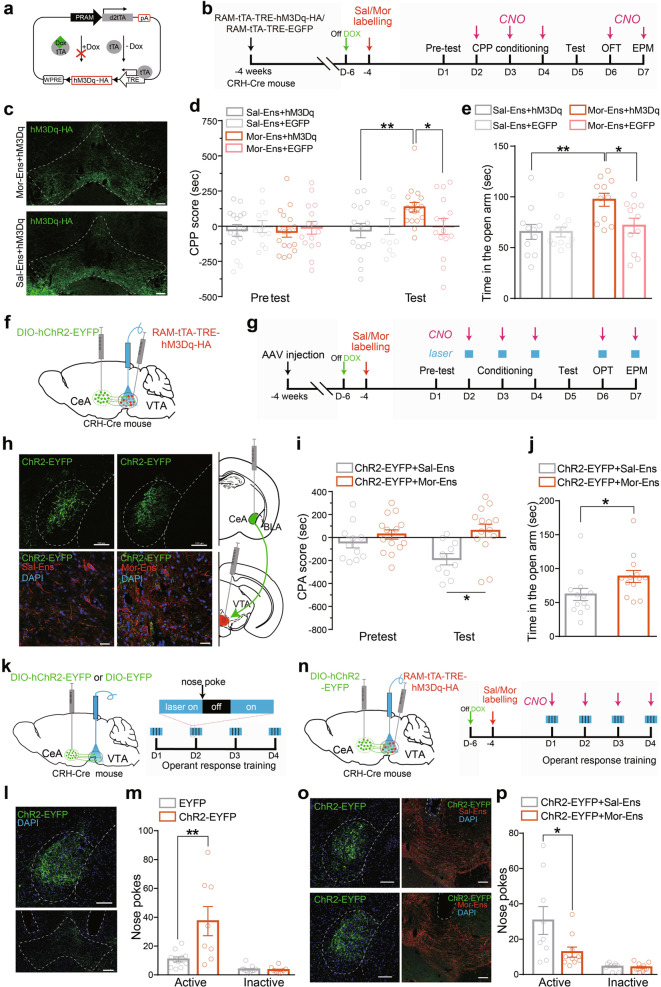
Chemogenetic activation of Mor-Ens in VTA alleviates the negative effect induced by activating CRH^CeA→VTA^ terminals. **a** Schematic representation of *AAV-RAM-tTA-TRE-hM3Dq* vector. **b** Experimental process of the CNO-evoked CPP experiment. **c** Representative images of expression of hM3Dq-HA in VTA Sal-Ens and Mor-Ens. Scale bar, 100 μm. **d, e** Chemoactivation of Mor-Ens drives CPP and promotes anxiolytic behavior. Two-way RM ANOVA, F_groups × session_ (1, 38) = 7.365, *P* = 0.011, Sal-Ens+hM3Dq vs Mor-Ens+hM3Dq within test, *P* = 0.006; F_groups × session_ (1, 30) = 4.917, *P* = 0.034, Mor-Ens+hM3Dq vs Mor-Ens+EGFP within test, *P* = 0.029; F_groups × session_ (1, 24) = 0.0006, *P* = 0.9799, Sal-Ens+hM3Dq vs Sal-Ens+EGFP within test, *P* > 0.999 in (**d**). Mann–Whitney test, Sal-Ens+hM3Dq vs Mor-Ens+hM3Dq, *U* = 20, *P* = 0.0065; Mor-Ens+hM3Dq vs Mor-Ens+EGFP, *U* = 26, *P* = 0.0233; Sal-Ens+hM3Dq vs Sal-Ens+EGFP, *U* = 60, *P* = 0.9851 in (**e**). **f** Schematic representation of virus injection and optic-fiber implantation in the VTA. **g** Experimental process of the opto- and chemogenetic manipulated behavioral tests. **h** Representative images of CRH neurons in the CeA expressing *hChR2-EYFP* (top), and Sal-Ens or Mor-Ens in the VTA labeled with hM3Dq-HA (bottom). Green: EYFP; Red: HA; Blue: DAPI. Scale bars, top: 100 µm; bottom: 20 μm. **i, j** The effect of chemogenetic activation of Mor-Ens and optogenetic activation of CRH^CeA→VTA^ terminals on the CPA and EPM tests. Two-way RM ANOVA, F_groups × session_ (1, 24) = 2.257, *P* = 0.146, hChR2+Sal-Ens vs hChR2+Mor-Ens within test, *P* = 0.010 in (**i**); Mann–Whitney test, *U* = 37, *P* = 0.014 in (**j**). **k** Left: Schematic representation of virus injection and optic-fiber implantation in the VTA for negative reinforcement task. Right: Experimental process of the operant response training. **l** Representative images of ChR2-EYFP expression in CeA and the projective terminals in the VTA. The dashed white lines indicated the optic fiber trace. Green: EYFP, Scale bars, 100 µm. **m** Average number of nose pokes in 60-min session on day 4. Unpaired *t* test, *t* = 3.026, *df* = 17, *P* = 0.0076. In total, 8–11 mice/group. **n** Left: Schematic representation of virus injection and optic-fiber implantation in the VTA for negative-reinforcement task. Right: Experimental process of the optical and chemogenetic manipulated operant-response training. **o** Representative images of ChR2-EYFP in CeA, and hM3Dq-HA in VTA. The dashed white lines indicated the optic-fiber trace. Green: EYFP; Red: HA; Blue: DAPI. Scale bars, 100 µm. **p** Average number of nose pokes in 60-min session on day 4. Unpaired *t* test, *t* = 2.215, *df* = 17, *P* = 0.0407. In total, 9–10 mice/group. *P < 0.05, **P < 0.001. Data are presented as mean ± SEM.

To verify the interplay of the stress and reward systems in vivo, *CRH-ires-Cre* mice were infected with *AAV-RAM-tTA-TRE-hM3Dq-HA* in the VTA to express hM3Dq in Mor- or Sal-Ens (Fig. 5a–c and Supplementary Fig. 11a–c). Chemogenetic activation of Mor-Ens, but not Sal-Ens during place conditioning, resulted in the preference for the CNO-paired chamber, and increased the time spent in the open arms (Fig. 5d, e), without affecting locomotor activity and the entries in the open arms (Supplementary Fig. 11d, e). These data are consistent with the results obtained with E-SARE-Cre^ERT2^/TAM system, that activation of Mor-Ens in VTA induced CPP and anxiolytic effect.

To assess whether activation of Mor-Ens could alleviate the negative effect driven by enhanced CRH^CeA→VTA^ transmission, *CRH-ires-Cre* mice were bilaterally infected with *AAV-DIO-hChR2-EYFP* in the CeA, and *AAV-RAM-tTA-TRE-hM3Dq-HA* in the VTA (Fig. 5f–h). We found that chemogenetic activation of Mor-Ens, but not Sal-Ens during the conditioning sessions, inhibited the development of conditioned aversion induced by optically activating the CRH^CeA→VTA^ terminals (Fig. 5i), and significantly increased the exploration time in the open arms in EPM test, whereas it had no effect on locomotor activity in open-field test (Fig. 5j and Supplementary Fig. 11f). These data indicate that the dysfunction of Mor-Ens, caused by enhanced CRH^CeA→VTA^ inputs, is required for the development of conditioned aversion of opiate withdrawal.

To confirm the negative effect of CRH^CeA→VTA^ terminal manipulation, an operant response to avoid optical activation of CRH^CeA→VTA^ terminals was performed. Mice expressing ChR2-EYFP or EYFP in CRH neurons in the CeA learned to terminate optogenetic activation of CRH^CeA→VTA^ terminals by nose pokes in operant chambers (Fig. 5k). The ChR2-EYFP-expressing group showed more active nose pokes to terminate laser stimulation than the EYFP-expressing group over three daily training sessions, resulting in less time of laser stimulation (Fig. 5l, m and Supplementary Fig. 12a, b). These results suggest that the activity of CRH^CeA→VTA^ terminals induces negatively reinforced response. While chemogenetic activation of Mor-Ens, but not Sal-Ens during the training sessions, inhibited the establishment of active avoidance of the optical activation of the CRH^CeA→VTA^ terminals (Fig. 5n–p and Supplementary Fig. 12c), indicating the specific modulation of CRH^CeA→VTA^ and Mor-Ens circuit is required for the development of negative reinforcement.

### CRHR1 in Mor-Ens mediates the inhibitory inputs from CRH^CeA→VTA^ neurons, and the negative effect developed during opiate withdrawal

**Fig. 6:**
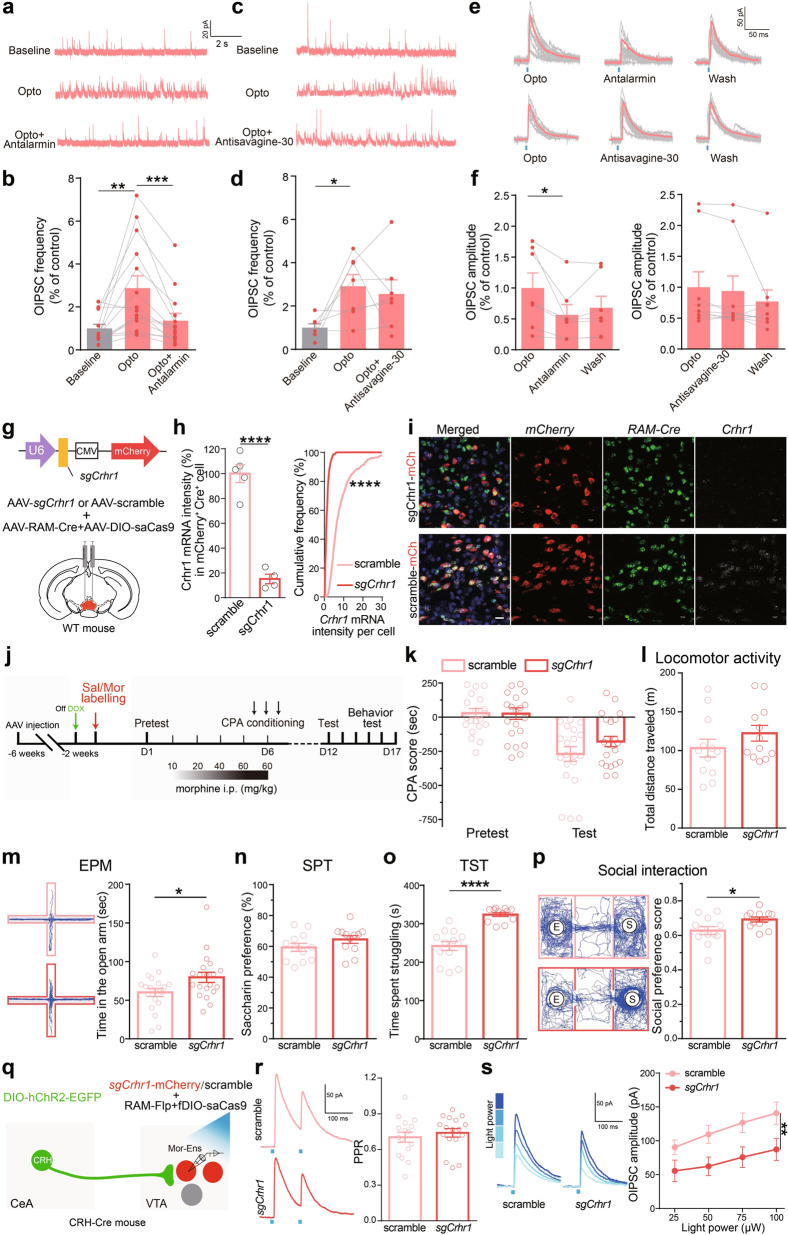
Pharmacological and genetic interventions of CRHR1 signaling attenuate the inhibitory inputs from CRH^CeA→VTA^, and the negative effect during opiate withdrawal. **a–d** Representative traces (**a, c**) and quantification of the IPSC frequency (**b, d**) in Mor-Ens following optical stimulation in the presence of antalarmin or antisavagine-30. Paired *t* test, Baseline vs Opto, *t* = 3.493, *df* = 13, *P* = 0.0044; Opto vs antalarmin, *t* = 4.482, *df* = 13, *P* = 0.0006 in (**b**). Baseline vs Opto, *t* = 2.909, *df* = 6, *P* = 0.0207; Opto vs antisavagine-30, *t* = 0.5294, *df* = 6, *P* = 0.6155 in (**d**). **e, f** Representative traces (**e**) and quantification of O-IPSC amplitudes (**f**) in Mor-Ens in the presence of antalarmin or antisavagine-30. Paired *t* test, Opto vs antalarmin, *t* = 2.920, *df* = 6, *P* = 0.0266; Opto vs antisavagine-30, *t* = 1.973, *df* = 8, *P* = 0.0840. **g** Schematic representation of the virus injection. **h** Quantification of the fluorescent intensity of *Crhr1* mRNA in mCherry^+^Cre^+^ cells. *sgCrhr1*: 419 cells from 4 mice; *scramble*: 596 cells from 5 mice. Unpaired *t* test, *t* = 9.632, *df* = *7*, *P* < 0.0001; Two-sample KS test, *P* < 0.0001. **i** Representative images of *Crhr1*, *Cre*, and *mCherry* ISH probes in *sgCrhr1* or *scramble* groups. Blue: DAPI; Green: Cre; Red: mCherry; Gray: *Crhr1*. Scale bar, 20 μm. **j–p** The effect of deletion of *Crhr1* in Mor-Ens on negative effect during morphine withdrawal. Experimental process of the behavioral assays (**j**). Morphine-withdrawal-induced CPA test (**k**), locomotor activity (**l**), EPM (**m**), SPT (**n**), TST (**o**), and social-interaction test (**p**). Two-way RM ANOVA, F_groups × session_ (1, 41) = 1.677, *P* = 0.2025, scramble vs *sgCrhr1* within test, *P* = 0.203 in (**k**). Mann–Whitney test or unpaired *t* test, scramble vs *sgCrhr1*, (**l**): *U* = 51, *P* = 0.2415; (**m**): *U* = 126, *P* = 0.0460; (**n**): *U* = 47, *P* = 0.1600; (**o**): *t* = 6.212, *df* = 23, *P* < 0.0001; (**p**): *U* = 36, *P* = 0.0387. **q** Schematic representation of optical stimulation and ensemble recordings. **r** Representative traces of PPR in response to optical stimulation (473 nm, 2-ms pulse width at 100-ms interval) and quantification of PPR values in *sgCrhr1* and scramble groups. Unpaired *t* test, *t* = 0.5813, *df* = 30, *P* = 0.5654. **s** Representative traces and quantification of the amplitudes of OIPSCs in Mor-Ens in response to different laser intensities (*n* = 16 neurons from five mice in *sgCrhr1* group, *n* = 16 neurons from six mice in scramble group). Two-way ANOVA, F_(3, 124)_ = 3.967, *P* = 0.0097. *P < 0.05, **P < 0.01, ***P < 0.001, ****P < 0.0001. Data are presented as mean ± SEM.

Since optostimulation might trigger neuropeptide corelease from the neuronal terminals [37], we then assessed the potential role of CRH–CRH-receptor signaling in the inhibitory CRH^CeA→VTA^ inputs onto the Mor-Ens. CRH^CeA→VTA^ terminals in the slice containing VTA were activated with optical stimulation. The frequency and amplitude of optical-evoked inhibitory postsynaptic currents (O-IPSCs) in Mor-Ens were significantly reduced by CRHR1 antagonist antalarmin; however, CRHR2 antagonist antisauvagine-30 had no effect on O-IPSCs in Mor-Ens (Fig. 6a–f). These data demonstrated that CRHR1-, but not CRHR2-mediated CRH signaling contributed to the inhibitory inputs onto the morphine ensembles, revealing a critical role of CRH signaling at plasticity remodeling of CeA CRH neurons–Mor-Ens circuit.

To determine whether CRHR1 signaling in Mor-Ens mediates the development of negative effect during opiate withdrawal, we constructed an AAV vector carrying sgRNA targeting *Crhr1* to achieve CRISPR–Cas9-mediated deletion of *Crhr1*. Mice were infected with *AAV-sgCrhr1-mcherry*, *RAM-tTA-TRE-Cre*, and Cre-dependent *AAV-DIO-saCas9* in the VTA. The possible effects of CRISPR-mediated off-site mutagenesis were excluded (Supplementary Fig. 13a–c). Single-molecule fluorescence in situ hybridization of *Crhr1* indicated that *Crhr1* mRNA levels in Mor-Ens infected with *sgCrhr1* (mCherry^+^Cre^+^) were dramatically decreased, as compared with control *sgRNA* group (Fig. 6g–i), demonstrating specific knockdown of *Crhr1* in Mor-Ens. CRHR1 deletion in either Mor-Ens or Sal-Ens showed no significant effect on conditioned aversion (Fig. 6j, k and Supplementary Fig. 14a, b) and locomotor activity (Fig. 6l and Supplementary Fig. 14c), and saccharin preference (Fig. 6n and Supplementary Fig. 14d) during opiate withdrawal. However, CRHR1 deletion in Mor-Ens, but not in Sal-Ens increased the entry time in the open arm (Fig. 6m and Supplementary Fig. 14f), the struggling time in tail suspension test (TST), and social-preference score during opiate withdrawal (Fig. 6o, p and Supplementary Fig. 14e, g), suggesting that CRHR1 of Mor-Ens contributes to the development of the negative effect, including anxiety, depression, and reduced sociability during opiate withdrawal.

To assess the role of CRHR1 signaling of Mor-Ens in CeA inhibitory tone following chronic morphine administration, *CRH-ires-Cre* mice were infected with *AAV-DIO-hChR2-EYFP* in the CeA, and *RAM-tTA-TRE-Flp*, *AAV-fDIO-saCas9*, and *AAV-sgCrhr1* in the VTA. O-IPSCs of Mor-Ens in response to the activation of CRH^CeA→VTA^ terminals were recorded, and the data from Cas9^−^ neurons were excluded by post-recording single-cell PCR (Supplementary Fig. 13d). CRHR1 deletion in Mor-Ens significantly decreased O-IPSC amplitude, while it had no effect on PPR (Fig. 6q–s), indicating that postsynaptic CRHR1 modulates the inhibitory inputs from CRH^CeA→VTA^ neurons to Mor-Ens. These results demonstrate that CRH–CRHR1 signaling contributes to the synaptic and functional remolding of the circuit of inhibitory CeA CRH inputs and Mor-Ens following chronic morphine administration, which mediates the negative effect during opiate withdrawal.

## Discussion

An increase in the mesolimbic dopamine is the commonality for positive reinforcement of addictive drugs. Previous studies have shown that optically activated VTA dopaminergic neurons are sufficient to trigger compulsive taking in mice, and evoke synaptic plasticity in the NAc associated with drug addiction [38]. Morphine acts via binding to opiate receptors to inhibit the VTA GABAergic neurons and thus increase dopamine levels [39]. Using the immediate early gene promoter-triggered labeling, we characterized activity-associated ensembles of VTA neurons encoding a positive-reward experience of initial morphine exposure. Activation of these neurons attenuates the negative effect such as conditioned aversion and anxiety during opiate withdrawal (Fig. 2). Following chronic morphine administration, the excitability of CeA→VTA-projective CRH neurons was upregulated, and the inhibitory inputs to the dopaminergic ensembles recruited by initial morphine exposure were increased to mediate the negative effect during morphine withdrawal.

VTA is a heterogeneous region that may play distinct roles in modulating reward and aversion based on connectivity to different upstream and downstream [13, 40, 41]. VTA DA neurons are active at baseline and there is spontaneous dopamine release in NAc as measured by voltammetry in behaving animals [10, 42]. Of note, the percentage of DA component was higher in NAc-projecting Mor-Ens than that in Sal-Ens. Optogenetic activation of Mor-Ens induces more DA release in NAc than Sal-Ens assessed by DA sensor (Fig. 1), consistently with previous studies demonstrating that opioid administration increases DA release in NAc by in vivo microdialysis [43]. Recent studies showed that VTA DA neurons activated by nicotine project to the NAc and maintain reinforcement. Inhibiting the VTA-to-amygdala DA neurons promotes anxiety [44]. Here, we found that VTA DA Mor-Ens sent more collaterals to NAc, and was selectively inhibited by CRH neurons in the CeA. The specific CeA–VTA Ens circuit was required for the development of reinforcement, conditioned aversion, and anxiety. These results indicate that DA neurons are heterogeneous in anatomically and functionally distinct subnetworks that might be recruited by different psychostimulants.

Increased probability of GABA release and GABAergic potentiation after chronic morphine treatment attenuates the rewarding effects of opioids [45–47], and Mor-Ens might receive diversity inhibitory inputs from VTA local or long-range circuits. Inhibitory synaptic inputs on TH^+^ Mor-Ens were increased, while in TH^−^, Mor-Ens was slightly decreased during morphine withdrawal (Fig. 2a–f and Supplementary Fig. 4e–g), indicating the different inhibitory innervation of TH^+^ and TH^−^ Ens during morphine withdrawal. The enhanced CRH^CeA→VTA^ GABAergic tone to the Mor-Ens following chronic morphine administration as a neuronal mechanism underlies the negative effect during opiate withdrawal. Considering the possible connection of the ensembles with local GABAergic neurons, the enhanced long-range inhibitory inputs might inhibit VTA GABAergic neurons, and then decrease the inhibitory inputs to TH^−^ Ens.

The CRH neurons are the predominant peptidergic neurons in the CeA, which also function as the main output of the nucleus [48]. Chemogenetic activation or inhibition of CeA CRH neurons bidirectionally modulates anxiety-like behavior, and mediates conditioned flight [49, 50]. VTA-innervating CRH neurons have been shown to be located in the bed nucleus of the stria terminals (BNST), PVN, and CeA [51–53]. In the current study, we found that CRH neurons in the CeA make monosynaptic connections with the VTA Mor-Ens (Fig. 4d–s). Optical inhibition of CRH^CeA→VTA^ terminals prevented the conditioned-place aversion and anxiety during opiate withdrawal (Fig. 3), indicating a specific role of CRH^CeA→VTA^ neurons in mediating the negative effect. Neurons in the BNST are thought to possess primarily a GABA phenotype, while glutamatergic neurons within the BNST also innervate the VTA [54], indicating the innervation of VTA from BNST might involve both inhibitory and excitatory mechanisms. Furthermore, CeA-projecting BNST cells were concentrated in the anterolateral (AL) and anteroventral (AV) sectors of BNST [55]. Whether CRH^BNST→VTA^ and CRH^BNST→CeA^ are coordinated in the modulation of Mor-Ens needs to be assessed in the future.

CRH produces an enhancement of IPSCs in dopaminergic neurons of the substantia nigra and VTA [56]. Intra-VTA CRH administration decreases the DA release in NAc responding to reward [57, 58]. However, optical activation of CRH neurons in CeA triggers the corelease of GABA and other neurotransmitters, such as protein kinase C δ and somatostatin, or even glutamatergic transmission with CRH [53, 59]. Hence, direct fast synaptic connections and indirect slow neuromodulatory action from CRH^CeA→VTA^ projections may jointly regulate the function of VTA Mor-Ens during morphine withdrawal. Genetic disruption of the CRHR1 in mice eliminates the negative effect of morphine withdrawal [60], while deletion of CRHR1 in midbrain dopaminergic neurons increases anxiety-like behavior and reduces DA release in the prefrontal cortex [61]. These data indicate that CRH-CRHR1 pathway exerts distinct effects on aversive or anxiogenic behaviors in drug-dependent versus drug-naive animals. Additionally, genetic dissection of CRHR1-expressing cells revealed that glutamatergic and dopaminergic systems mediate anxiogenic and anxiolytic effects of CRHR1, and might function in a concerted but antagonist manner to keep emotional responses to stressful situations [61]. Our results showed that CRHR1 antagonist and CRHR1 deletion attenuated the O-IPSCs in response to activation of CRH^CeA→VTA^ terminals (Fig. 6a–f, q–s), indicating that CRH–CRHR1 pathway facilitates GABAergic tone onto the Mor-Ens in the VTA following chronic morphine administration. Deletion of CRHR1 in Mor-Ens alleviated anxiety, depression, and impaired social interaction, while it did not affect conditioned aversion during opiate withdrawal (Fig. 6j–p), suggesting that CeA GABAergic and CRH connections with Mor-Ens might exert not exactly the same effects on negative effect.

In this study, we investigated into the underlying CRH-mediated inhibitory neurotransmission and plasticity changes of the neuronal ensembles encoding drug-reward experience. The inhibitory inputs of CRH^CeA→VTA^ neurons onto Mor-Ens were enhanced following chronic morphine administration, and thus promoted the negative effect by dysregulating the function of those ensembles. We proposed that activation of ensembles encoding a drug-reward experience offers a potential node for alleviating the negative effect driven by enhanced inhibitory inputs from CeA, and thus preventing opiate dependence. Expression of immediate early genes has been used to identify diverse experience-defined ensembles, and drive distinct behavior reinforcement [62, 63]. In our studies, the activation of morphine-recruited ensembles labeled by either the enhanced *Arc* or *c-fos* promoter is sufficient to elicit appetitive responses and positive reinforcement. Considering the heterogeneous composition of the ensembles, phasic firing pattern in DA neurons, delayed DA release, and GABA activation [64, 65] should be considered for the specific neurotransmission and plasticity modulation in the ensembles. Therefore, specifically labeling opiate-recruited dopaminergic and GABAergic ensembles will facilitate the identification of the functional neuronal subtype in the heterogeneous VTA neurons involved in the diverse information processing of the opiate reward in future. Nevertheless, our data suggest that modulating CRH GABAergic circuit between CeA and VTA may be a potential strategy for treating the negative reinforcement produced by drug withdrawal.

## Materials and methods

### Animals

All animal procedures were conducted in accordance with the animal care guidelines approved by the Animal Care and Use Committee of the School of Basic Medical Sciences of Fudan University and the guidelines of the National Institutes of Health. *CRH-ires-Cre B6(Cg)-Crhtm1(cre)Zjh/J* mice (012704) were obtained from Jackson Lab (CA, USA), and were bred on a *C57 BL/6* background for more than six generations. Male offspring at 6–12 weeks of age were used in the experiments, and randomly assigned to groups. Genotypes were determined by polymerase chain reaction (PCR) of mouse tail DNA samples. About 6–9-weekold *C57 BL/6* male mice were purchased from Shanghai Laboratory Animal Center (CAS, Shanghai, China). The mice used for experiments were housed in plastic cages with disposable bedding on a standard 12-h light/dark cycle with food and water available ad libitum. Experiments were performed during the light phase.

### Viral vectors

To obtain the neuronal ensembles that respond to specific stimuli, we used a synthetic promoter, the enhanced synaptic activity-responsive element (E-SARE), which was constructed by multiplexing the 104-bp SARE enhancer fragment of SARE-ArcMin, also expressed the drug-inducible recombinase ER^T2^Cre^ERT2^ under the control of E-SARE [66, 67]. The *pAAV-E-SARE-Cre*^*ERT2*^ plasmid was kindly provided by Prof. Haruhiko Bito (The University of Tokyo, Japan). To generate the *pAAV-RAM-d*_*2*_*TTA-pA*::*TRE-Cre-WPRE-pA* plasmids, we replaced *EGFP* in *pAAV-RAM-d2TTA::TRE-EGFP-WPRE-pA* (Addgene: 84469) with the *Cre* sequence obtained by PCR from *pAAV-Cre-GFP* (Addgene: 68544). To generate the *pAAV-RAM-d*_*2*_*TTA-pA::TRE-Flp-WPRE-pA* plasmids, we replaced *EGFP* in *pAAV-RAM-d2TTA::TRE-EGFP-WPRE-pA* with the *Flp* sequence obtained by PCR from *pAAV-TRE-DIO-Flp* (Addgene: 118027). To generate the plasmids *pAAV-RAM-d*_*2*_*TTA-pA::TRE-hM3D(Gq)-HA-WPRE-pA*, we replaced *EGFP* in *pAAV-RAM-d2TTA::TRE-EGFP-WPRE-pA* with *hM3D(Gq)* obtained by PCR from the templates of *pAAV-hSyn-hM3Dq-mCherry* (Addgene: 50474). All the AAV vectors were serotyped with AAV_9_ and packaged by Obio Technology Co., Ltd (Shanghai, China). *AAV-EF1α-DIO-hM3Dq-mCherry, AAV-EF1α-DIO-hM4Di-mCherry, AAV-EF1α-DIO-mCherry, AAV-EF1α-DIO-EGFP, AAV-Retro-EF1α-DIO-EYFP, AAV-Retro-hSyn-tdTomato, AAV-CAG-DIO-saCas9, AAV-CAG-fDIO-saCas9, AAV-EF1α-DIO-hChR2(H134R)-EYFP*, and *AAV-EF1α-DIO-hChR2(H134R)-mCherry* were purchased from Taitool Bioscience Co., Ltd. (Shanghai, China). *AAV-EF1α-DIO-hM3Dq-HA*, and *AAV-EF1α-DIO-eNpHR3.0-EYFP* were purchased from the University of North Carolina (Vector Core, NC, USA). *RV-ENVA-deltaG-dsRed*, *AAV-hSyn-DA4.4, AAV-DIO-H2B-EGFP-TVA*, *AAV-DIO-RVG, AAV-CMV-sgRNA (Scramble)-mCherry*, and *AAV-CMV-sgRNA(Crhr1)-mCherry* were purchased from BrainVTA Co., Ltd. (Wuhan, Hubei, China).

### Stereotaxic surgery and laser stimulation

Mice were anesthetized by 2% isoflurane for surgery in the stereotactic instrument (Stoelting, Kiel, WI, USA). Microinjections were performed using 33-gauge needles connected to a 10-μL microsyringe (Hamilton, Nevada, USA). The intended stereotaxic coordinates for the ventral tegmental area (VTA) were − 3.2-mm AP; ML ±0.9-mm ML (with an angle of 10° from the middle to the lateral); −4.4-mm DV; for CeA were − 1.30-mm AP; ±2.70-mm ML; −4.40-mm DV. Each site was injected with 0.5 μL of purified and mixed AAV (10^12^ IU/mL) with a slow-injection rate of 0.1 μl/min. About 200-μm-diameter optic-fiber cannula (0.37 NA) was implanted above the VTA with −4.20-mm DV coordinate. Light was delivered by a 473-nm or 594-nm laser diodes (Brain-King, China). The light intensity at the fiber tip was measured using a light sensor (Thorlabs, Newton, NJ, USA). An 8~10-mW laser pulse (20-Hz frequency, 5-ms duration) generated by a Master-8 pulse stimulator (AMPI, Jerusalem, Israel) was delivered through the embedded optical fiber in the VTA. The mice with off-target mCherry or EGFP location were excluded from analysis.

### Labeling-activated ensembles

To label the ensembles with the *E-SARE-Cre*^*ERT2*^ system, 10 mg/ml tamoxifen (Sigma-Aldrich) was prepared in a mixed oil solution (of nine parts corn oil and 1-part ethanol), and was injected intraperitoneally (i.p.) at the dose of 125 mg/kg. Mice were given morphine (10 mg/kg, i.p.) or equivalent volume of saline injection 24 h after TAM administration to label the ensembles. To label ensembles with the *RAM-tTA-TRE* system, mice were taken off doxycline (Dox) diet (40 mg/kg) 48 hrs before the injection of morphine or saline, and then kept back on Dox 12 h later. Mice were given at least five days to allow protein expression before the electrophysiological and behavior experiments.

### Pavlovian conditioning

Conditioned-place preference (CPP) was performed by unbiased procedures in the two-chamber apparatus with distinct tactile environments to maximize contextual differences (Med-Associates, St. Albans, VT, USA). Mice were randomly assigned to either the experimental or control group for next. The procedures consisted of pretest, and conditioning test. On day 1, mice were placed in the middle of the conditioning chambers and allowed to freely explore the entire apparatus for 20 min (pretest). The sessions were recorded by infrared-tracking instrument and the time spent in each chamber was determined. Mice that stayed in one chamber for more than 13 min were excluded from the experiment. On days 2, 3, and 4, mice received the intraperitoneal injection of CNO (2 mg/kg) or laser stimulation while confined to one of the chambers for 30 min (conditioning) and then received an equivalent volume of saline while confined to the other chamber for 30 min 6 h later. On day 5, mice were allowed to freely explore the entire apparatus for 20 min (test). The time spent in each chamber was recorded during the pretest and test sessions. The CPP score was defined as the time (in seconds) spent in the CNO-paired chamber minus the time spent in the saline-paired chamber.

Morphine-withdrawal-induced conditioned-place aversion (CPA) procedure: mice were allowed to freely explore both sides of the CPA apparatus for 20 min to assess their baseline place preference (pretest). Then, these mice received injections of morphine (i.p., b.i.d) at escalating doses (10, 20, 40, 60, and 60 mg/kg for five consecutive days) in their home cages. About 9 h after each of the last 3 morphine injections, when somatic withdrawal symptoms were induced, mice were confined in one chamber (withdrawal-paired) of the apparatus for 30 min. Mice were reexposed to the apparatus for 20 min six days after the last conditioning trial. The CPA score was defined as the time (in seconds) spent in the withdrawal-paired chamber minus the time spent in the other side of the chamber. For chemogenetic manipulations, mice were given an injection of CNO (2 mg/kg, i.p.) 30 min prior to each of the CPA conditioning.

### Optical intracranial self-stimulation (ICSS)

Three weeks following virus infection, mice labeled with Mor-Ens or Sal-Ens were given six daily intracranial self-stimulation (ICSS) training sessions. Behavioral training and testing were performed in a mouse-operant apparatus (Med Associates) interfaced with optogenetic stimulation equipment (Newdoon Inc and Inper Tech, Hangzhou, Zhejiang, China). Each operant behavior chamber was equipped with a dim-light source and a nose-poke hole equipped with infrared photobeams connected to a computer. A 1-h fixed-ratio one (FR1) schedule was performed every training day. A nose poke in the target hole produced 2 s of 473-nm light (5 ms, 20 Hz, 10 mW). For drug-infusion studies, mice received injections of either flupenthixol (0.5 mg/kg, Tocris, Bristol, UK) or saline vehicle intraperitoneally 30 min prior to ICSS sessions. For negative-reinforcement procedures [68], mice were placed into the chamber and delivered continuous 473-nm (5 ms, 20 Hz, 10 mW) optical stimulation with an interstimulus interval of 1 s. The mice were trained on a FR1 training schedule, in which each active nose poke produced a 20-s laser shut-off and the terminals were not optogenetically activated.

### Open-field test (OFT) and elevated-plus-maze (EPM) test

An activity-monitor system (43.2-cm length × 43.2-cm width × 30.5 cm height, Med-Associates, USA) was used to detect the locomotor activity. Each mouse was placed in the center of the open field and allowed to explore freely for 30 min. The center was defined as a square of 50% of the total OFT area. The entries in the center zone, time in the center zone, and total distance traveled were recorded and analyzed. For chemogenetic manipulations, mice were given an intraperitoneal injection of CNO (2 mg/kg) 30 min prior to behavior test.

The elevated plus maze consisted of four arms (34.5-cm length × 6.3-cm width × 19.5-cm height) and a center platform placed 75 cm above the floor. Two of the arms had 20 cm high dark walls (closed arms), and two had 0.8-cm high ledges (open arms). The arms were angled at 90° to each other. The apparatus was placed in a quiet and dimmed room. Mice were placed in the center and allowed to freely explore the maze for 6 min. Their behaviors were recorded with a camera located above the maze and analyzed by Etho Vision XT 8.5 video-tracking program (Leesburg, VA, USA). The arms were cleaned with water and dried between each test to ensure the absence of olfactory cues.

### Saccharin-preference test (SPT) and tail suspension test (TST)

The morphine-withdrawal-induced decrease in the preference for saccharin is thought to reflect anhedonia-like states. Animals were habituated with two bottles of water for 36 h, followed by one bottle of 0.1% (w/v) saccharin solution and one bottle of water. Bottle positions were switched every 12 h. After training, mice were restricted from water for 12 h and then exposed to a choice of two bottles for 1.5-h test. The saccharin preference was calculated by dividing the total consumption of saccharin by the total consumption of both water and saccharin. For chemogenetic manipulations, mice were given an intraperitoneal injection of CNO (2 mg/kg, i.p.) 30 min prior to the test session.

For tail-suspension test, the tail of the mouse was fixed with a tape at a distance of 1 cm from the end, and then suspended upside down on a crossbar 15–20 cm from the floor without any accessible surface for 6 min. After a period of agitation, there was intermittent immobility, indicating a state of depression. Videos were taken for trials to record the cumulative time of struggling and immobility within 6 min. The box was cleaned with 75% ethanol and dried between trials.

### Social-preference test

As previously described [69], the mouse was put in a three-chambered arena (60 × 40 × 22 cm). The luminosity was around 7 lux. Each mouse in the experiment group and the control group was placed in the center chamber for 10 min for habituation period. Then in the test period, two round-wire cages, which permitted nose contact between bars but prevented extensive physical contact, were placed in the lateral chambers. One cage was empty, the other had an unfamiliar mouse (three days for habituation, 10 min per day). The unfamiliar mouse needs to be changed every two trials, and the mouse as the stranger should be placed at the different cage between trials to prevent the place preference. The cage was cleaned with 75% ethanol and dried between trials. Videos were taken for every session and the time of exploration (approach, sniffing, and rearing within 2 cm from the cage) of the stranger mouse and the empty cage by the test mouse was recorded. The social-preference score = the time interact with the stranger mouse/the total exploration time.

### Behavioral-spectrum analysis

Mice infected with *AAV-DIO-hM3Dq-mCherry* combined with *AAV-E-SARE-Cre*^*ERT2*^ were labeled with morphine-positive or saline-positive ensembles. These mice received intraperitoneal injections of morphine (i.p.) twice daily in their home cages for five consecutive days (with escalating dose as described above). Nine hours after the last morphine injection, mice received an injection of CNO (2 mg/kg, i.p.) and withdrawal symptoms were recorded for 20 min by a camera at 30 FPS. Somatic symptoms (walking, stilling, grooming, sniffing, digging, hanging, rearing, and tremors) were manually quantified by an individual blinded to the group allocation. *t*-Distributed stochastic neighbor embedding (*t*-SNE) was used to evaluate the changes of the whole spectrum of behaviors. We performed *t*-SNE on the ethogram matrix composed of eight behaviors (walk, dig, still, hang, groom, rear, sniff, and tremor) during spontaneous withdrawal. Each column represents one behavior. Each row represents one mouse. The variable values are the total duration or times of each behavior during 20 min of spontaneous withdrawal. The main setting parameters for *t*-SNE: Output dimensionality = 2, Perplexity parameter = 5, Number of iterations = 500.

### In vivo optic-fiber recording

An optical fiber with an outer diameter of 200 or 400 μm and 0.50 NA (Inper Tech, Hangzhou, Zhejiang, China) was implanted into the VTA or NAc of mice as described above. Fluorescence signals were recorded using a Fiber Photometry system equipped with a 470- and 410-nm excitation lasers (Inper Tech). Optical stimulation (594 nm, 10 mW, 5-ms pulse, 20 Hz, 2-s duration) was delivered every 20 s for 10–15 trials through the fiber implanted in the VTA. The other fiber was used for recording in NAc. About 470 nm excites fluorescence from the genetically encoded indicator of DA sensor (DA4.4) for measuring dopamine release [70]. About 410 nm serves as a control for movement and bleaching. Fluorescence signals were recorded using a script provided by Inper Tech. The 470- and 410-nm signals were independently processed and normalized to baseline signals to determine ∆*F*/*F*, where ∆*F*/*F* = (*F*–*F*_*0*_)/*F*_*0*_ and *F*_*0*_ is the mean value of the integral of the prestimulus signal (2 s) [71]. Fluorescence signals were sampled at 50 Hz for a single channel. The baseline correction and motion-correction strategies were used in the process of data analysis. The 410-nm signal was used as reference signal to control for motion-related artifacts. The 410-nm trace was scaled using least-squares regression to minimize the difference between the 410- and 470-nm signal and subtracted from the 470 trace to generate the corrected 470-nm signal [72]. The PolynomialFitted correction was used to reduce the photobleaching effect caused by long-term recording. Data were analyzed using MATLAB, ∆*F*/*F* values are presented as heatmaps and average plots, with the shaded area indicating the standard error of the mean. The mice with off-target fiber-tip location were excluded from analysis.

### Brain-slice preparation and electrophysiological recordings

Acute coronal sections (300 μm) containing the CeA or VTA were prepared as previously described [73, 74]. Briefly, the mice were anesthetized deeply by isoflurane (3–5% induction, 1.5–2% maintenance) and then perfused with cold NMDG-based artificial cerebrospinal fluid (ACSF). Brains were quickly removed, sliced with the vibratome (HM650V, Thermo Scientific, Wilmington, MA, USA), and incubated in NMDG-based ACSF at 32–34 °C for 10 min. Individual cells were visualized by a BX51WI microscope (Olympus, Tokyo, Japan) equipped with Rolera Bolt CCD camera (QImaging, Surrey, BC, Canada). Whole-cell voltage-clamp recordings were performed in oxygenated-standard ACSF with an EPC-10 amplifier and Patchmaster software (HEKA Elektronik, Lambrecht/Pfalz, Germany). A modified intracellular solution (127.5 mM cesium methanesulfonate, 7.5 mM CsCl, 10 mM HEPES, 2.5 mM MgCl_2_, 4 mM Na_2_ATP, 0.4 mM Na_3_GTP, 10 mM sodium phosphocreatine, 0.6 mM EGTA, and 0.4% biocytin, pH 7.25, 290 mOsm) was used to adjust the reversal potential of the *γ*-aminobutyric acid A-receptor (GABA_A_R) response. Excitatory postsynaptic current (mEPSC) events were recorded in the presence of 2 μM TTX and a GABA_A_R blocker (bicuculline methiodide, 10 μM), at a holding potential of −60 mV. Spontaneous miniature-inhibitory postsynaptic current (mIPSC) events were recorded in the presence of 2 μM TTX, 10 μM D-APV, and 20 μM CNQX, at a holding potential of +10 mV.

For optogenetic stimulation, collimated light (2–3 mW) from an LED (X-site 110) controlled by a TTL input from a digital signal output (HEKA Instruments) was delivered through the epifluorescence pathway of the microscope and fed into a 60 × water-immersion lens. To confirm the function of eNpHR3.0, 594-nm light pulses (0.5 s in duration with 0.5-s interval) were delivered, while neuronal firing was evoked by a 100 pA of somatic current injection. The activation of hChR2 was assessed by current clamp. Cells were stimulated with 473-nm light pulses at 20 Hz to invoke reliable generation of action-potential (AP) firing. A current-step protocol (from -100 to +200 pA, with a 10-pA increment) was run and repeated. The after-hyperpolarization potential (AHP), threshold, and input resistance were sampled following the first single action-potential spike.

For light-evoked inhibitory postsynaptic current recordings, neurons were clamped at +10 mV. D-APV (100 mM) and CNQX (20 mM) were added to the intracellular solution to block excitatory currents. ChR2-expression fibers were excited with a train of 473-nm light pulses (20 Hz, duration 5 ms), and the postsynaptic responses were recorded for 1 min. For evoked IPSC recordings, the duration of the pulse was 1–2 ms. Once a stable evoked response was achieved, a baseline was recorded (5 min) and then the perfusion was switched to ACSF containing 600 μM antalarmin (Sigma-Aldrich, A8727, St Louis, WA, USA) or 300 μM antisauvagine-30 (Tocris, #2071) for 20-min recording. Recordings with R_s_ > 30 MΩ were excluded from statistical analysis. Electrophysiological data were analyzed off-line with Clampfit 10.3 (Molecular Devices, Union City, CA, USA) or Mini Analysis Program (Synaptosoft Inc, Fort Lee, NJ, USA).

### Rabies tracing

Trans-synaptic tracing studies were carried out as previously described [75] with minor modifications. For rabies tracing, mice were infected with *AAV-E-SARE-Cre*^*ERT2*^, AAV*-DIO-H2B-EGFP-TVA*, and *AAV-DIO-RVG* in the VTA. Three weeks later, mice were injected with TAM (125 mg/kg, i.p.) 24 h before ensemble labeling. A single dose of morphine (10 mg/kg) or saline was injected intraperitoneally for labeling. *RV-ENVA-deltaG-dsRed* was injected into the VTA at the same coordinates on the next day. Mice were housed in the ABSL-3 facility for 7 days before the histological processing. For rabies-tracing analysis, consecutive brain slices (50-μm thickness) selected from every fifth slice were collected throughout the brain, and dsRed^+^ input neurons and starter cells were manually counted by an experimenter, blind to the experimental condition.

### RNAscope ISH

As previously described [74], the frozen brain tissue was sliced into 10-μm coronal sections and mounted onto Colorfrost Plus slides (Thermo Scientific). Slices were incubated with hydrogen peroxide for 10 min at RT, and then performed target retrieval and proteolysis using RNAscope^®^ 2.5 Universal Pretreatment Reagents (ACD: 322380). SmFISH for all genes examined, *Cre* (#402551), *Crh* (#316091), *dsRed* (#481361-C2), *Crhr1* (#418011-C3), and *mCherry* (#431201-C2) performed hybridization for 2 h. After hybridization, we used RNAscope^®^ Multiplex Fluorescent Detection Kit v2 (ACD: 323110) to amplify the signal. Images were acquired with 20 × objective (Nikon A1, Tokyo, Japan) . IOD in *mCherry*- and *Cre-*positive cells or *Crh*-positive neurons was analyzed by Image-Pro Plus 6.0 (Media Cybernetics, Rockville, MD, USA). The observer analyzing the expression of *Crhr1* and *Crh* in neuronal ensembles was blinded to the group allocation.

### Immunohistochemistry

Mice were anesthetized and perfused as described previously. The brain was isolated, fixed in 4% PBS-buffered PFA, and dehydration in sucrose solutions (30%) at 4 °C. The 50-μm frozen coronal slices were prepared by Leica CM3050 S Cryostat (Buffalo Grove, IL, USA). Slices were incubated in primary antibody in block buffer (10% goat serum and 0.2% Triton X-100) over night. After washing by PBS, the slices were incubated in secondary antibody for 1 h and DAPI for 5 min at room temperature. The slices were mounted in mounting medium (Sigma). Primary antibodies used were anti-HA (H6908-0.5 ml, Sigma, 1:500), anti-c-Fos (sc-52, Santa Cruz, Dallas, TX, USA), anti-CRH (ab8901, Abcam, Cambridge, MA, USA), anti-TH antibodies (MAB318, Merck, 1:1000), and DAPI (D9534, Sigma). Secondary antibodies were anti-mouse 488 (711-545-150, Jackson ImmunoResearch, 1:1000) and anti-rabbit Cy3 (115-165-116, Jackson ImmunoResearch, 1:500). Images were acquired on a Nikon-A1 confocal microscope (Tokyo, Japan) using a 20 × objective lens. Biocytin-backfill slices were incubated with Cy3- or Alexa Fluor-488-conjugated streptavidin (Jackson ImmunoResearch, 016-160-084, 016-540-084, 1:1000) to secondary antibody solution.

### Ensemble axonal projection collateralization

Sample fixation and slides were processed as described above in the “Immunohistochemistry” section. Coronal sections (30-µm thickness) were analyzed with Image J by applying equal thresholds to all images and measuring the integrated density (IntDen) contained in a 250 × 250-pixel zone for each target brain area. The intensity of mCherry-expressing fibers could be assessed for major VTA Sal-Ens or Mor-Ens anatomical target. Data were presented as a relative-intensity ratio normalized with Sal-Ens in the NAc, mPFC, amygdala, and LHb.

### Analysis of off-target effects of CRISPR-mediated genome editing

sgRNAs were designed using online CRISPR tools (http://crispr.mit.edu/ and http://chopchop.cbu.uib.no/), sgRNA *(5*′*-ACTCCACCGACCGTCTGCGCAAGTGGA-3*′*)* targeting the exon of *Crhr1*, and scramble sgRNA *(5*′*-GCACTACCAGAGCTAACTCA-3*′*)*. To investigate the specificity of CRISPR–Cas9-mediated knockout of *Crhr1*, online CRISPR tools (https://www.benchling.com/crispr/) were used to predict the off-target sites. Neuro-2A (N2A) cells were cultured in Dulbecco’s modified Eagle’s medium (Thermo Fisher, MA, USA) supplemented with 10% fetal bovine serum (Thermo Fisher). The *U6-sasgCrhr1* cassette was cloned into the *psaCas9-NLS-P2A-Puro-T2A-EGFP* backbone from commercial resource (BrainVTA Co., Ltd). Cells were transfected with Lipofectamine 3000 (Thermo Fisher, L3000008). Genomic DNA was extracted and PCR amplification was performed using the following primers: *Crhr1* on-target primer forward, 5′-CATCTCGGCTTTCATCCTGC-3′; reverse, 5′-CCTGCTGTGACAATGAGCTC-3′; *Crhr2* off-target forward, 5′-GCTGGTCATCTTGGGTAGGA-3′reverse, 5′-GGTACCCCACTGGGAGTTTT-3′; *1700017B05Rik* off-target forward, 5′-AAAGAGACGCTCCTTCACCA-3′; reverse, 5′-GCTCCAGCCACCAGTAAGAG-3′; *Pdlim2* off-target forward, 5′-CCAGGAAAAGGGAAAGAAGG-3′; reverse, 5′-AGCCAGGGCTACACAGAGAA-3′. PCR products were digested with T7 endonuclease and analyzed by DNA gel electrophoresis. Cleavage efficiency was calculated as cleavage efficiency = 1– [(1– fraction cleaved)1/2], where fraction cleaved = sum of cleaved-band intensities/(sum of the cleaved and parental band intensities).

### Single-cell RT-PCR

Patch pipettes were pulled (P-2000, Sutter Instrument, Novato, CA, USA) to a resistance of 4–6 MΩ. After electrophysiology recording, the cytoplasm was collected into a 0.2-mL PCR tube containing 20 U Ribonuclease inhibitor (Takara, Japan). The presence of mRNA coding saCas9 for the recorded cells was determined by reverse transcription in the presence of random hexamer. The first-round PCR contained 20 cycles and the product (2 μL) was used as the template for the second-round PCR that contained 45 cycles. The final products were visualized by electrophoresis in agarose gels (2.0%). The specific primers for saCas9 gene were custom designed and synthesized (Genewiz, Suzou, Jiangsu, China), 5′-GAAATACGTGGCCGAACTGC-3′ sense and 5′-GACAGGTCCACCTTCTTGGG-3′ antisense (first round); 5′-GACCTGTACAACGCCCTGAA-3′ sense and 5′-TTCAGCCGGTTGAAGATAGCGAT-3′ antisense (second round). Primers were used to generate a PCR product of 506 b.p. in size.

### Quantification and statistical analysis

Data were analyzed with SPSS 22 software (IBM, Armonk, NY, USA), and plotted by Graphpad Prism. Our sample sizes were based on our previous research [74, 76, 77]. The normality test of the data was performed by Shapiro–Wilk test and the homoscedasticity was performed by F test. The nonnormalized data were analyzed with nonparametric test. Comparisons between groups were made by student’s *t* test (unpaired, two tailed), paired *t* test, Mann–Whitney U test, *χ*^2^ test, and one-way ANOVA or two-way ANOVA. Two-sample *Kolmogorov–Smirnov* test was used for cumulative frequency and amplitude plot analysis. Behavior results of CPA and CPP were analyzed by two-way RM ANOVA. The Bonferroni’s post hoc analysis was performed after one-way ANOVA or two-way ANOVA. Statistical significances were represented as *P < 0.05, ** *P* < 0.01, ***, *P* < 0.001 and ****, *P* < 0.0001. All data are presented as mean ± SEM.

## Supplementary information

Supplemental material
