# Supplementary material for: CRHCeA→VTA inputs inhibit the positive ensembles to induce negative effect of opiate withdrawal
Source: Mol Psychiatry. 2021 Oct 12;26(11):6170–86. doi: 10.1038/s41380-021-01321-9 (PMC8760059; doi:10.1038/s41380-021-01321-9)
Supplement: Supplementary file 1 — Supplemental material [file 41380_2021_1321_MOESM1_ESM.docx]

**CRH^CeA→VTA^ inputs control the positive ensembles to modulate negative affect of opiate withdrawal**

*Supplementary Material*

**Figure S1. Time window for tamoxifen-induced ensembles-labeling and the verification of labeling specificity. Related to Figure 1.**

**Figure S2. The distribution in VTA, cell composition and terminal density of Sal-Ens and Mor-Ens. Related to Figure 1.**

**Figure S3. The effects of activation of Mor-Ens on conditioned place preference (CPP), open field test (OFT), elevated plus maze (EPM) and saccharin preference tests (SPT). Related to Figure 1.**

**Figure S4. Synaptic transmission in Sal-Ens and Mor-Ens. Related to Figure 2.**

**Figure S5. The effect of CNO on opiate withdrawal. Related to Figure 2.**

**Figure S6. Chemogenetic manipulation of Mor-Ens on negative affect during opiate withdrawal. Related to Figure 2.**

**Figure S7. The synaptic transmission and the electrophysiological properties of CRH^CeA→VTA^ neurons following chronic morphine administration. Related to Figure 3.**

**Figure S8. The effects of optogenetic manipulation of CRH^CeA^**^→^**^VTA^ terminals on the locomotor activity. Related to Figure 3.**

**Figure S9. Identification of the TH^+^ starters and quantification of the inputs on Sal- and Mor-Ens. Related to Figure 4.**

**Figure S10. Identification and quantification of Mor-Ens and Sal-Ens captured by E-SARE and RAM systems. Related to Figure 4.**

**Figure S11. Activation of Mor-Ens has no effect on the locomotor activity of mice in which the CRH^CeA^**^→^**^VTA^ terminals were optical activated. Related to Figure 5.**

**Figure S12**. **Establishment of nose-poking behavior in the negative reinforcement task. Related to Figure 5.**

**Figure S13. Analysis of the off-target effects of CRISPR-mediated genome editing, and the expression of *saCas9* in patching neurons by single-cell RT-PCR. Related to Figure 6.**

**Figure S14. The effect of CRHR1 deletion in Sal-Ens on the behavioral tests during opiate withdrawal. Related to Figure 6.**

**Supplemental Table1. Key reagents and software.**


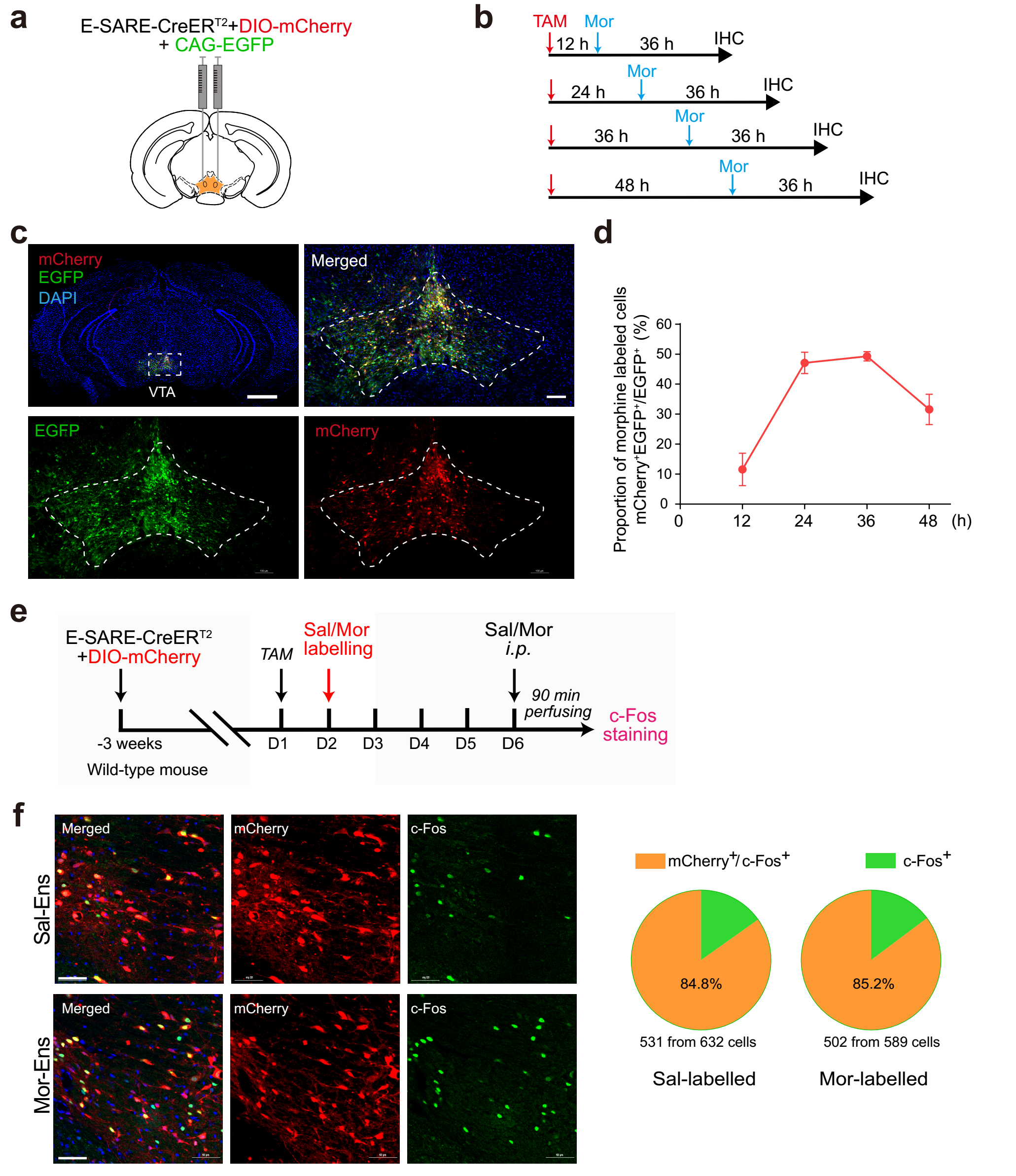


**Figure S1. Time window for tamoxifen-induced ensembles-labeling and the verification of labeling specificity. Related to Figure 1.**

**a-d** Time window for the tamoxifen-induced ensembles-labeling. Schematic representation of the virus injection (**a**) and the experimental procedure (**b**). The mice infected with *AAV-DIO-mCherry*, *AAV-E-SARE-Cre^ERT2^*, and *AAV-CAG-EGFP* (as infection control) in the VTA were intraperitoneal (i.p.) injected with morphine (10 mg/kg) in their home cages at 12 hrs, 24 hrs, 36 hrs, and 48 hrs after tamoxifen injection (125 mg/kg, i.p.). Mice were sacrificed 36 hrs after morphine injection. **c** Representative images of the VTA from the mice injected with morphine 24 hrs after tamoxifen injection. **d** Quantification of the labeling efficiency at different time point (12-48 hrs) after tamoxifen injection in the VTA. Green: EGFP, Blue: DAPI, Red: mCherry. Scale bar, left: 1000 μm; right: 200 μm. n = 3 mice, per time point. **e, f** Experimental procedure of ensembles labeling. Mice were [intraperitoneal](https://cn.bing.com/dict/search?q=Intraperitoneal&FORM=BDVSP6&mkt=zh-cn) [injected](https://cn.bing.com/dict/search?q=administration&FORM=BDVSP6&mkt=zh-cn) with TAM (125 mg/kg), and morphine (10 mg/kg) or saline was injected 24 hrs later. Verification of the ensembles labeling by c-Fos staining 5 days later. (**e**). Representative images and quantification of Mor-Ens or Sal-Ens co-localized with c-Fos^+^ (**f**). Red: mCherry, Green: c-Fos, Blue: DAPI, Scale bar, 50 μm. n = 4-5 mice per group. Data are presented as mean ± SEM.


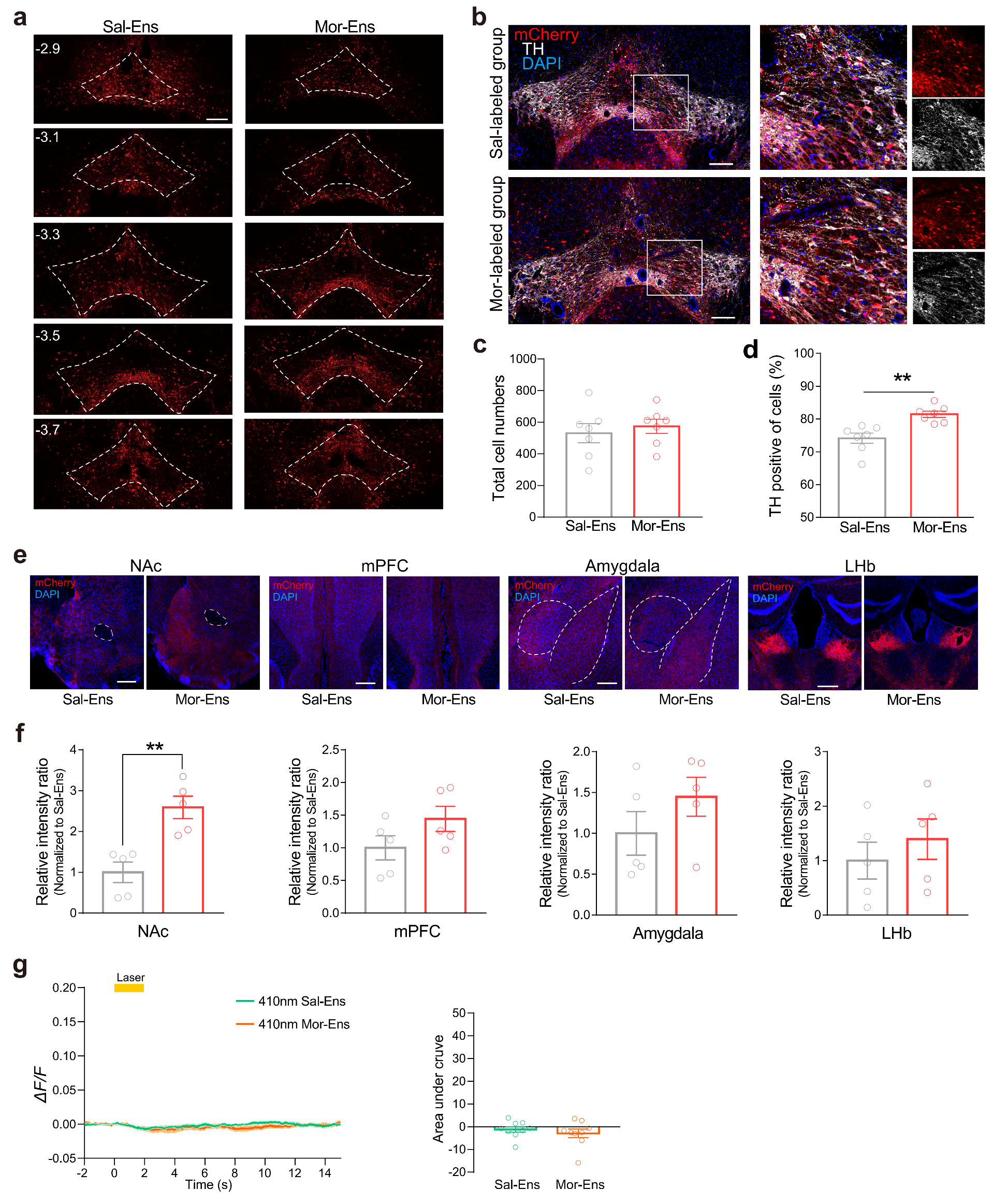


**Figure S2. The distribution in VTA, cell composition and terminal density of Sal-Ens and Mor-Ens. Related to Figure 1.**

**a** Representative images Mor-Ens and Sal-Ens in the VTA across the anterior posterior axis (-2.9 ~ -3.8 mm from bregma). Red: mCherry. Scale bar, 200 μm. **b** TH staining in the slice containing VTA. Red: mCherry; Grey: TH; Blue: DAPI. Scale bar, 100 μm. **c** Total cell number labeled (mCherry^+^) in Mor-Ens and Sal-Ens (n = 7 mice per group). Unpaired t test, t = 0.586, df = 12, *P* = 0.5688. **d** Percentage of TH^+^ cells in labeled VTA ensembles (-3.2 ~ -3.5 mm from bregma). 1320 TH^+^ cells from 1785 Sal-Ens and 1958 TH^+^ cells from 2397 Mor-Ens group, n = 7 mice per group. Unpaired t test, *t* = 4.033, *df* = 12, *P* = 0.0017. **e** Representative images of the mCherry*^+^* axon terminals of the ensembles in LHb, NAc, Amygdala, and mPFC. Scale bar, 100 μm or 200 μm. **f** Relative fluorescence intensity of mCherry terminals. n = 5 mice per group, Mann Whitney U test, NAc: *U* = 0, *P* = 0.0079; mPFC: *U* = 6, *P* = 0.4316; Amygdala: *U* = 7, *P* = 0.3095; LHb: *U* = 9, *P* = 0.5476. **g** Average *ΔF*/*F* % and the area under the curve of 410 nm fluorescence in response to optical stimulation of Sal-Ens or Mor-Ens. 9 mice per group. ***P* < 0.01. Data are presented as mean ± SEM.


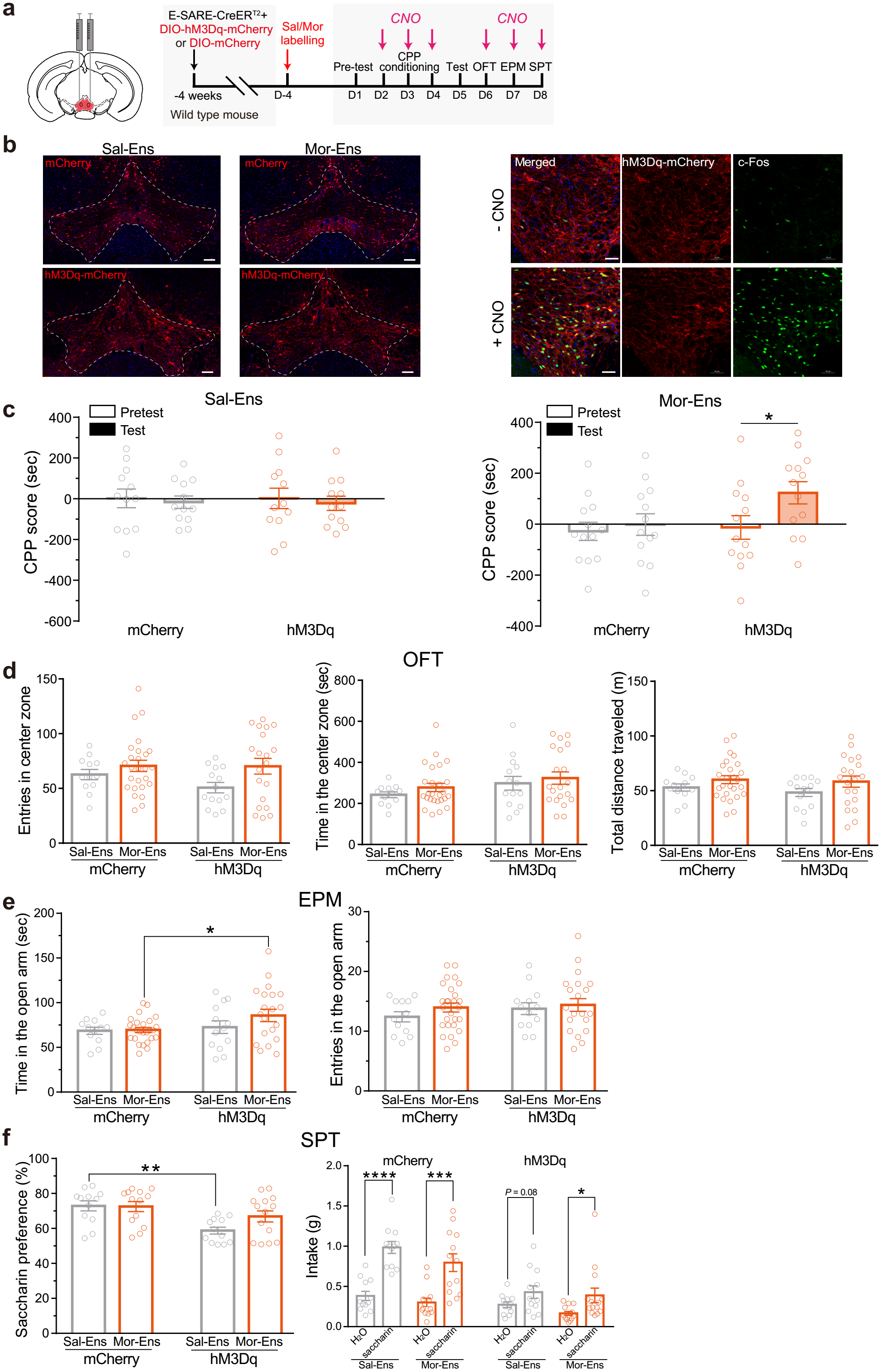


**Figure S3. The effects of activation of Mor-Ens on conditioned place preference (CPP), open field test (OFT), elevated plus maze (EPM) and saccharin preference tests (SPT). Related to Figure 1.**

**a** Schematic representation of the virus injection and behavioral tests. **b** Representative images of the hM3Dq-mCherry or mCherry expression and activation of the ensembles by CNO in Mor-Ens*.* Red: mCherry; Green: c-Fos. Scale bar, left: 100 μm; right: 50 μm. **c** CPP scores of Sal-Ens or Mor-Ens groups mice in the pre-test and test sessions. Wilcoxon matched-pairs signed rank test, Sal-Ens+mCherry: *P* = 0.8501. Paired t test, Sal-Ens+hM3Dq: *t* = 0.6136, *df* = 11, *P* = 0.6136; Mor-Ens+mCherry: *t* = 0.8322, *df* = 12, *P* = 0.4215; Mor-Ens+hM3Dq: *t* = 2.589, *df* = 12, *P* = 0.0237. **d** Effects of chemogenetic activation of Sal-Ens and Mor-Ens in the OFT. Entries in the center zone: Sal-Ens mCherry *vs* Sal-Ens hM3Dq, Mann Whitney test, *U* = 49.5, *P* = 0.0778; Sal-Ens hM3Dq *vs* Mor-Ens hM3Dq, Unpaired t test, *t* = 1.83, *df* = 34, *P* = 0.0761; Mor-Ens mCherry *vs* Mor-Ens hM3Dq, Mann Whitney test, *U* = 256.5, *P* = 0.9432. Time in the center zone: Sal-Ens mCherry *vs* Sal-Ens hM3Dq, Unpaired t test, *t* = 1.463, *df* = 24, *P* = 0.1566; Sal-Ens hM3Dq *vs* Mor-Ens hM3Dq, Mann Whitney test, *U* = 128, *P* = 0.6913; Mor-Ens mCherry *vs* Mor-Ens hM3Dq, Mann Whitney test, *U* = 217, *P* = 0.3493. Distance: Sal-Ens mCherry *vs* Sal-Ens hM3Dq, Mann Whitney test, *U* = 72, *P* = 0.5604; Sal-Ens hM3Dq *vs* Mor-Ens hM3Dq, Unpaired t test, *t* = 1.362, *df* = 34, *P* = 0.1938; Mor-Ens mCherry *vs* Mor-Ens hM3Dq, Mann Whitney test, *P* = 0.2608. **e, f** The effects of chemogenetic activation of Mor-Ens in the EPM (**e**) and SPT (**f**) tests. Mor-Ens mCherry *vs* Mor-Ens hM3Dq, Unpaired t test, time: *t* = 2.436, *df* = 44, *P* = 0.0190; entries: Mann Whitney test, *U* = 254, entries: *P* = 0.7364; Sal-Ens hM3Dq *vs* Mor-Ens hM3Dq, Mann Whitney test, *U* = 97, *P* = 0.2348; entries: Mann Whitney test, *U* = 119, *P* = 0.6954; Sal-Ens mCherry *vs* Sal-Ens hM3Dq, Unpaired t test, time: *t* = 0.491, *df* = 23, *P* = 0.6281; entries: Mann Whitney test, *U* = 65, *P* = 0.4907 in (**e**). Left: Mann Whitney test, Mor-Ens mCherry *vs* Mor-Ens hM3Dq: *U* = 60, *P* = 0.1373; Sal-Ens hM3Dq *vs* Mor-Ens hM3Dq: *U* = 50, *P* = 0.0826; Sal-Ens mCherry *vs* Sal-Ens hM3Dq: *U* = 19, *P* = 0.0014. right: Sal-Ens mCherry, H_2_O *vs* saccharin: *U* = 4, *P* < 0.0001; Unpaired t test, Mor-Ens mCherry, H_2_O *vs* saccharin, *t* = 4.065, *df* = 24, *P* = 0.0004; Sal-Ens hM3Dq, H_2_O *vs* saccharin, *t* = 1.819, *df* = 22, *P* = 0.0826; Mor-Ens hM3Dq, H_2_O *vs* saccharin, *t* = 2.371, *df* = 26, *P* = 0.0254 in (**f**).. **P* < 0.05, ***P* < 0.01, ****P* < 0.001, *****P* < 0.0001. Data are presented as mean ± SEM.


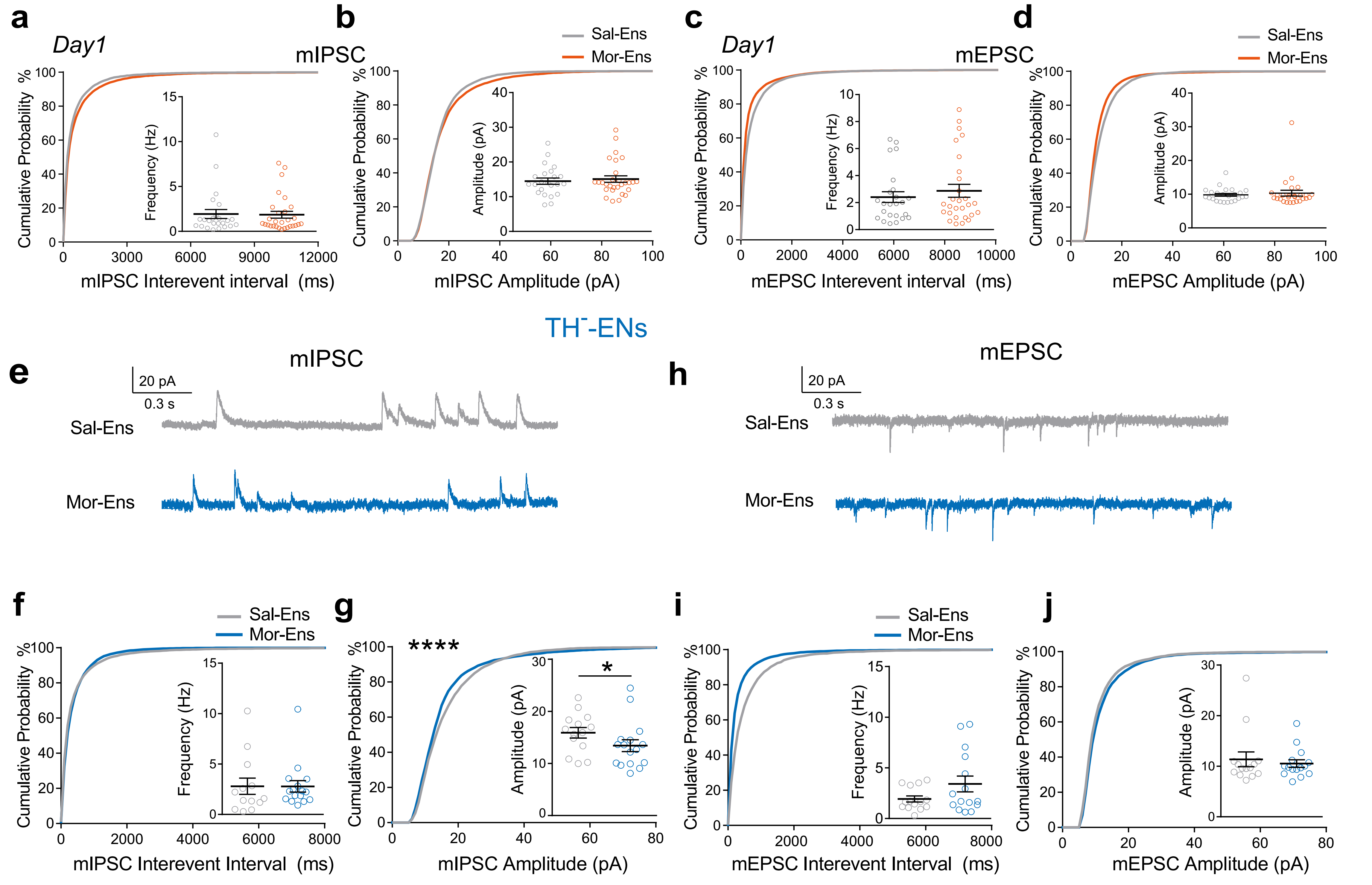


**Figure S4. Synaptic transmission in Sal-Ens and Mor-Ens. Related to Figure 2.**

**a-d** The cumulative probability and average amplitude and frequency of mIPSCs (**a, b**) or mEPSCs (**c, d**) recorded from Sal-Ens and Mor-Ens without escalating-dose of morphine exposure (n = 5 mice per group). Mann Whitney U test, *P* = 0.7569 for frequency and *P* = 0.9372 for amplitude in (**a, b**); *P* = 0. 3579 for frequency and *P* > 0.99 for amplitude in (**c, d**). **e-j** Representative traces (**e, h**), cumulative probability distribution, and average amplitude and frequency of mIPSCs (**f, g**) and mEPSCs (**i, j**) recorded from TH^-^-VTA ensembles after escalating-dose of morphine administration. Unpaired t test, frequency: *t* = 0.0135, *df* = 27, *P* = 0.9893 in (**f**); Mann-Whitney U test, amplitude: *U* = 62, *P* = 0.0383 in (**g**); Unpaired t test, frequency: *t* = 1.716, *df* = 27, *P* = 0.0976 in (**i**); amplitude: *t* = 0.527, *df* = 27, *P* = 0.6025 in (**j**). Two-sample KS test, interval: *P* < 0.0001 in (**g**). **P* < 0.05, *****P* < 0.0001. Data are presented as mean ± SEM.


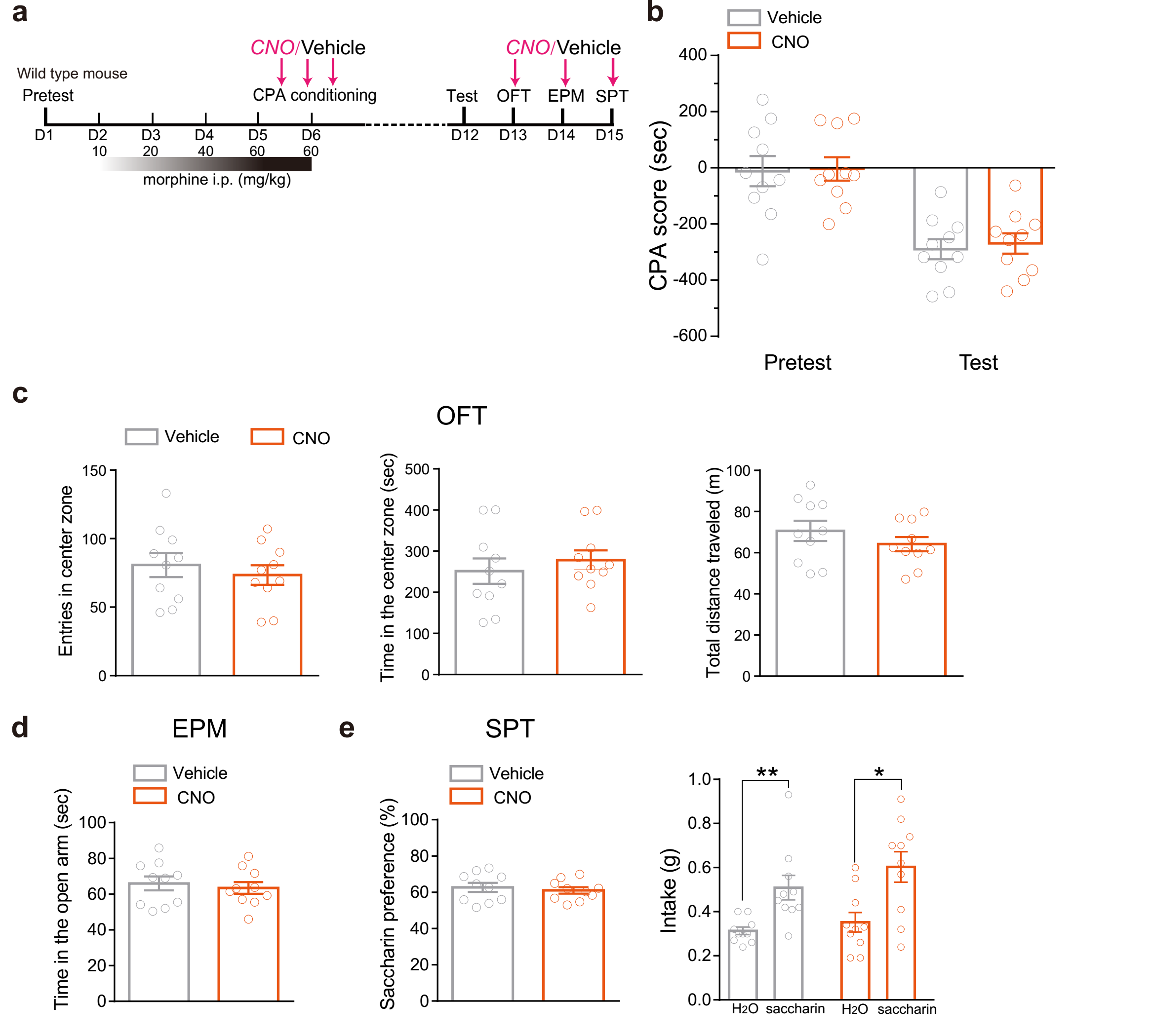


**Figure S5. The effect of CNO on opiate withdrawal. Related to Figure 2.**

**a** Experimental procedure of CNO injection and morphine withdrawal-induced behavioral tests. **b** CPA scores of vehicle or CNO groups in the pre-test and test sessions. Two-way RM ANOVA, F _groups × session_ (1, 18) = 0.0200, *P* = 0.8891, Vehicle *vs* CNO within test, *P* > 0.99. **c** Effects of CNO injection on the OFT. Entries in the center zone: *U* = 44, *P* = 0.6705; Time in the center zone: *U* = 42, *P* = 0.5787; Distance: *U* = 35, *P* = 0.2799; Mann Whitney U test. **d, e** Effects of CNO injection on the EPM (**d**) and SPT (**e**) tests. Mann Whitney U test, *U* = 46.5, *P* = 0.8115 in (**d**). Left: Mann Whitney U test, *U* = 43, *P* = 0.6305; right: Vehicle intake, H_2_O *vs* saccharin, Unpaired t test, *t* = 3.385, *df* = 16, *P* = 0.0033, CNO intake, H_2_O *vs* saccharin, Mann Whitney U test, *U* = 17.5, *P* = 0.0122 in (**e**). **P* < 0.05, ****P* < 0.001. Data are presented as mean ± SEM.


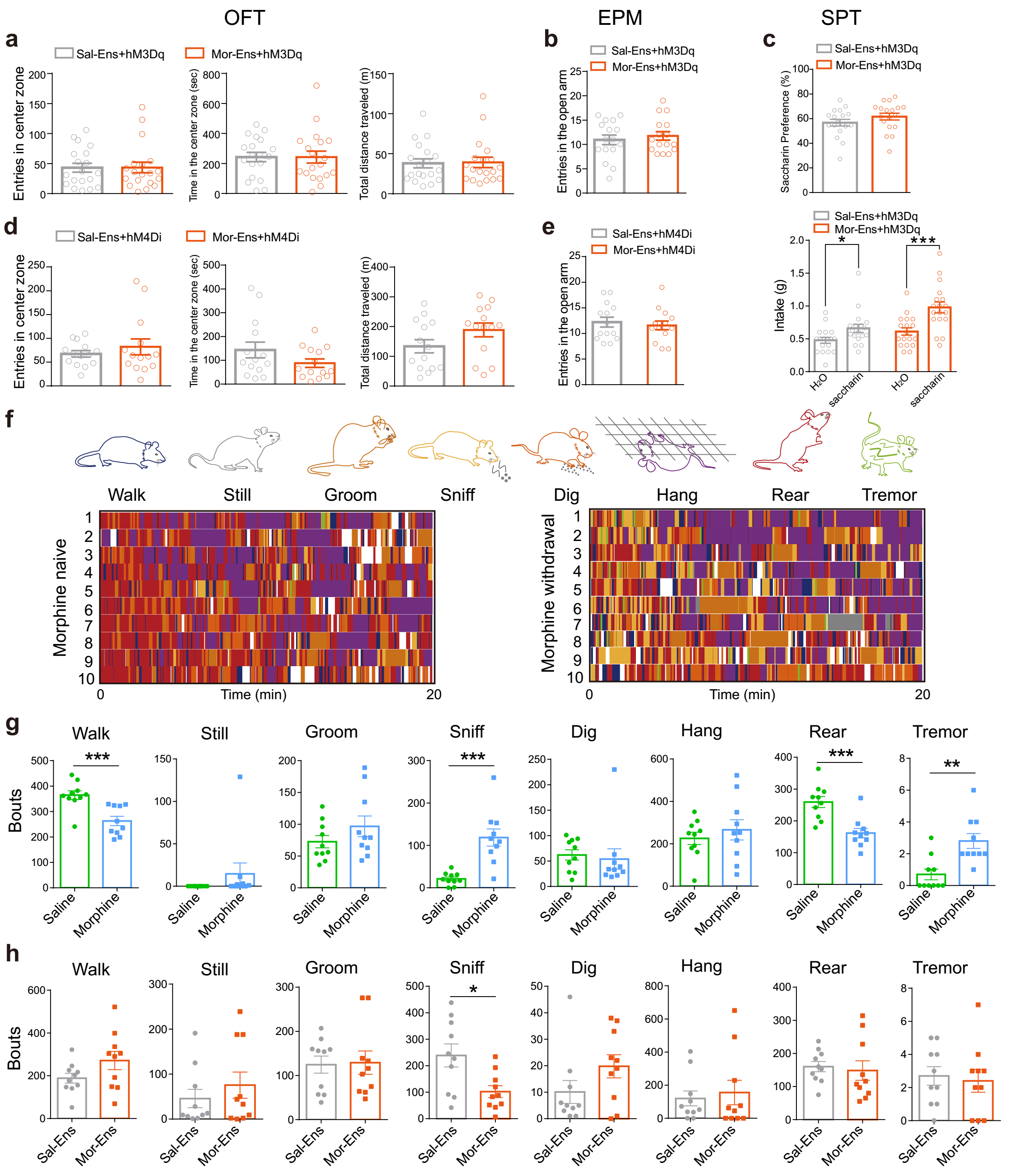


**Figure S6. Chemogenetic manipulation of Mor-Ens on negative affect during opiate withdrawal. Related to Figure 2.**

**a-c** Effects of chemogenetic activation of Sal-Ens and Mor-Ens during opiate withdrawal on the OFT (**a**), EPM (**b**) and SPT (**c**) tests. Mann Whitney U test. Entries in the center zone, *U* = 178, *P* = 0.9482; Time in the center zone, *U* = 166, *P* = 0.6861; Distance, *U* = 178, *P* = 0.9540 in (**a**); *U* = 121.5, *P* = 0.8157 in (**b**). Top: *U* = 119.5, *P* = 0.1829; down: Sal-Ens, H_2_O *vs* saccharin, *U* = 84, *P* = 0.0120, Mor-Ens, H_2_O *vs* saccharin, *U* = 57, *P* = 0.0005 in (**c**). **d, e** Effects of inhibition of Sal-Ens and Mor-Ens on OFT (**d**) and EPM (**e**). Entries in the center zone, Unpaired t test, *t* = 0.8189, *df* = 26, *P* = 0.4203; Time in the center zone, Unpaired t test, *t* = 1.482, *df* = 26, *P* = 0.1504; Distance, Mann Whitney U test, *U* = 67, *P* = 0.1636 in (**d**); Mann Whitney U test, *U* = 91.5, *P* = 0.7766 in (**e**). **f** Behavioral spectrums analysis of mice in saline (left) and morphine withdrawal (right) groups. (**g**) The analysis of eight behaviors in mice treated with saline or escalating-dose of morphine. Walk, *t* = 4.075, *P* = 0.0007; Still, *t* = 1.145, *P* = 0.2674; Groom, *t* = 1.278, *P* = 0.2175; Sniff, *t* = 4.682, *P* = 0.0002; Dig, *t* = 0.3610, *P* = 0.7223; Hang, *t* = 0.7205, *P* = 0.4805; Rear, *t* = 4.280, *P* = 0.0005; Tremor, *t* = 3.656, *P* = 0.0018. (**h**) The analysis of eight behaviors of the mice in which Sal-Ens and Mor-Ens were activated by CNO during withdrawal. Walk, *t* = 1.700, *P* = 0.1063; Still, *t* = 0.8368, *P* = 0.4137; Groom, *t* = 0.1377, *P* = 0.8920; Sniff, *t* = 2.788, *P* = 0.0121; Dig, *t* = 1.581, *P* = 0.1312; Hang, *t* = 0.4141, *P* = 0.6837; Rear, *t* = 0.3542, *P* = 0.7273; Tremor, *t* = 0.3390, *P* = 0.7386. **P* < 0.05, ***P* < 0.01, ****P* < 0.001. Unpaired t test. **P* < 0.05, ***P* < 0.01, ****P* < 0.001. Data are presented as mean ± SEM.


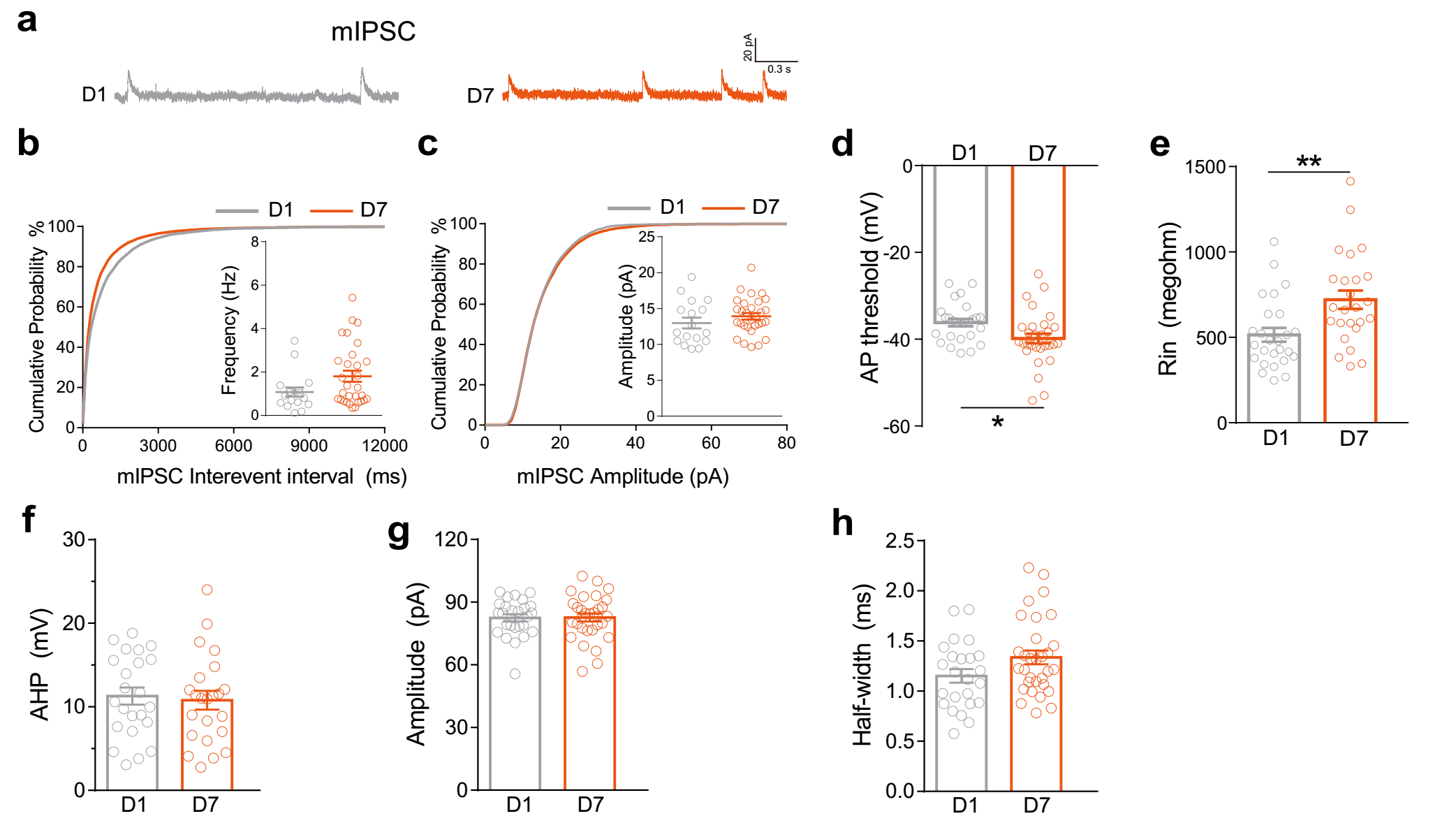


**Figure S7. The synaptic transmission and the electrophysiological properties of CRH^CeA→VTA^ neurons changed following chronic morphine administration. Related to Figure 3.**

**a-c** Representative traces (**a**), the cumulative probability, average amplitude and frequency of mIPSCs (**b, c**) recorded from CRH^CeA→VTA^ neurons. Unpaired t test, *t* = 1.916, *df* = 45, *P* = 0.0618 in (**b**). Mann Whitney U test, *U* = 199, *P* = 0.2217 in (**c**). KS test for cumulative probability distribution, interval: *P* < 0.0001, amplitude: *P* = 0.0192. **d-h** Quantification of the basic parameters of the APs in threshold (**d**), input resistance (Rin) (**e**), AHP (**f**), amplitude (**g**) and half-width (**h**). Unpaired t test, Threshold: *t* = 2.533, *df* = 55, *P* = 0.0142; Rin: *t* = 3.085, *df* = 49, *P* = 0.0033; AHP: *t* = 0.3306, *df* = 44, *P* = 0.7425; Amplitude: *t* = 0.0499, *df* = 55, *P* = 0.9604; Half-width: *t* = 1.887, *df* = 53, *P* = 0.0646. **P* < 0.05, ***P* < 0.01, *****P* < 0.0001. Data are presented as mean ± SEM.


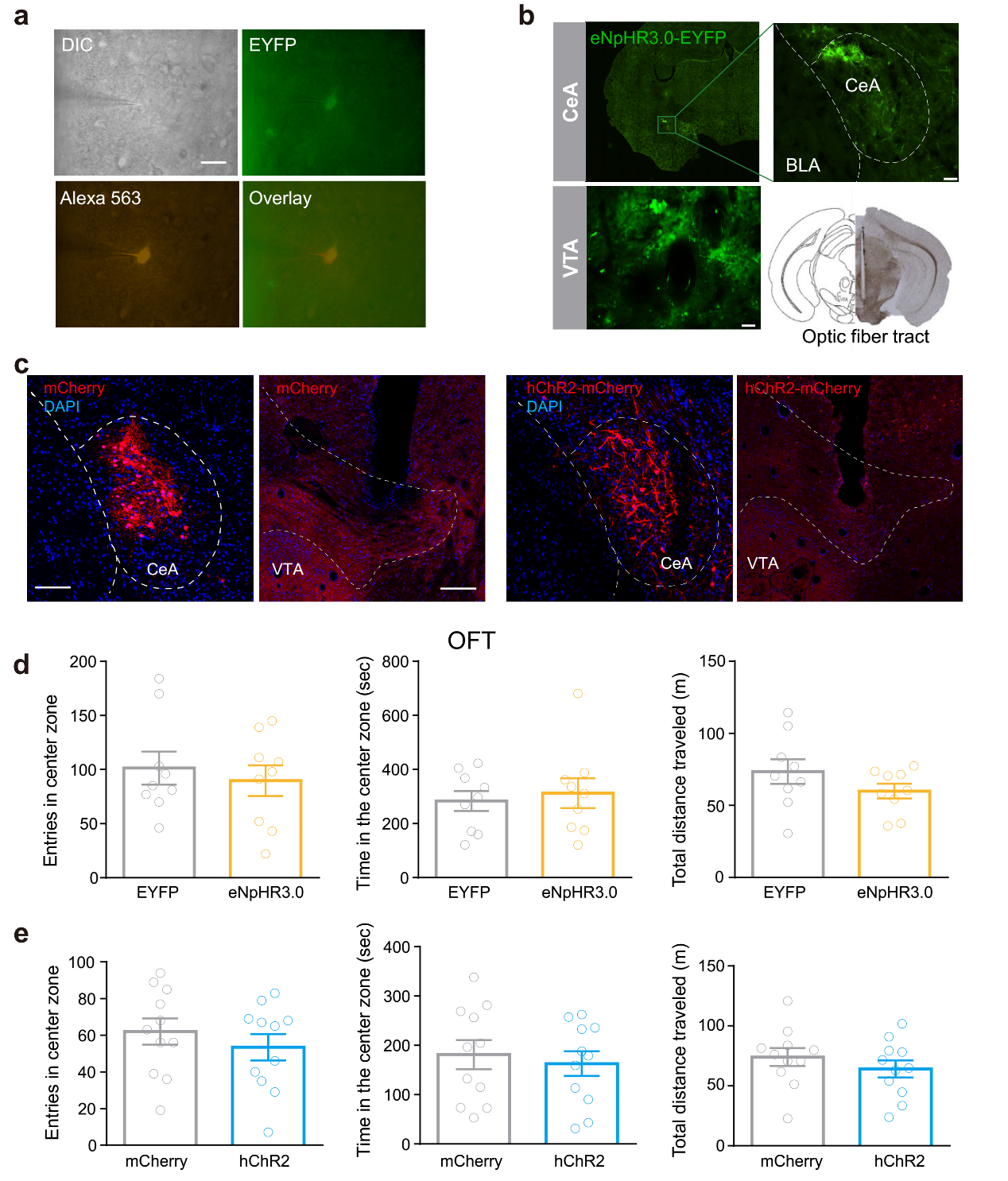


**Figure S8. The effects of optogenetic manipulation of CRH^CeA^**^→^**^VTA^ terminals on the locomotor activity. Related to Figure 3.**

**a** Representative image of the EYFP^+^ CRH neurons recorded in the CeA. Scale bar, 20 μm. **b** Representative images of CRH neurons expressing eNpHR3.0 (green) in the CeA and their axon terminals in the VTA. Green: EYFP. Scale bar, upper: 50 μm; bottom: 20 μm. **c** Representative image of CRH neuron expressing hChR2 (red) in the CeA and their axon terminals in the VTA. Red: mCherry. Scale bar, 100 μm. **d** The effect of optical inhibition of CRH^CeA→VTA^ terminals on the OFT during morphine withdrawal. For the time in the center zone, *t* = 0.4379, *df* = 16, *P* = 0.6673; for distance, *t* = 1.357, *df* = 16, *P* = 0.1936. **e** The effect of optical activation of CRH^CeA→VTA^ terminals on OFT. Unpaired t test, for entries in the center zone, *t* = 0.8432, *df* = 20, *P* = 0.4091; for the time in the center zone, *t* = 0.4712, *df* = 20, *P* = 0.6426; for distance, *t* = 0.9519, *df* = 20, *P* = 0.3525. Unpaired t test. Data are presented as mean ± SEM.


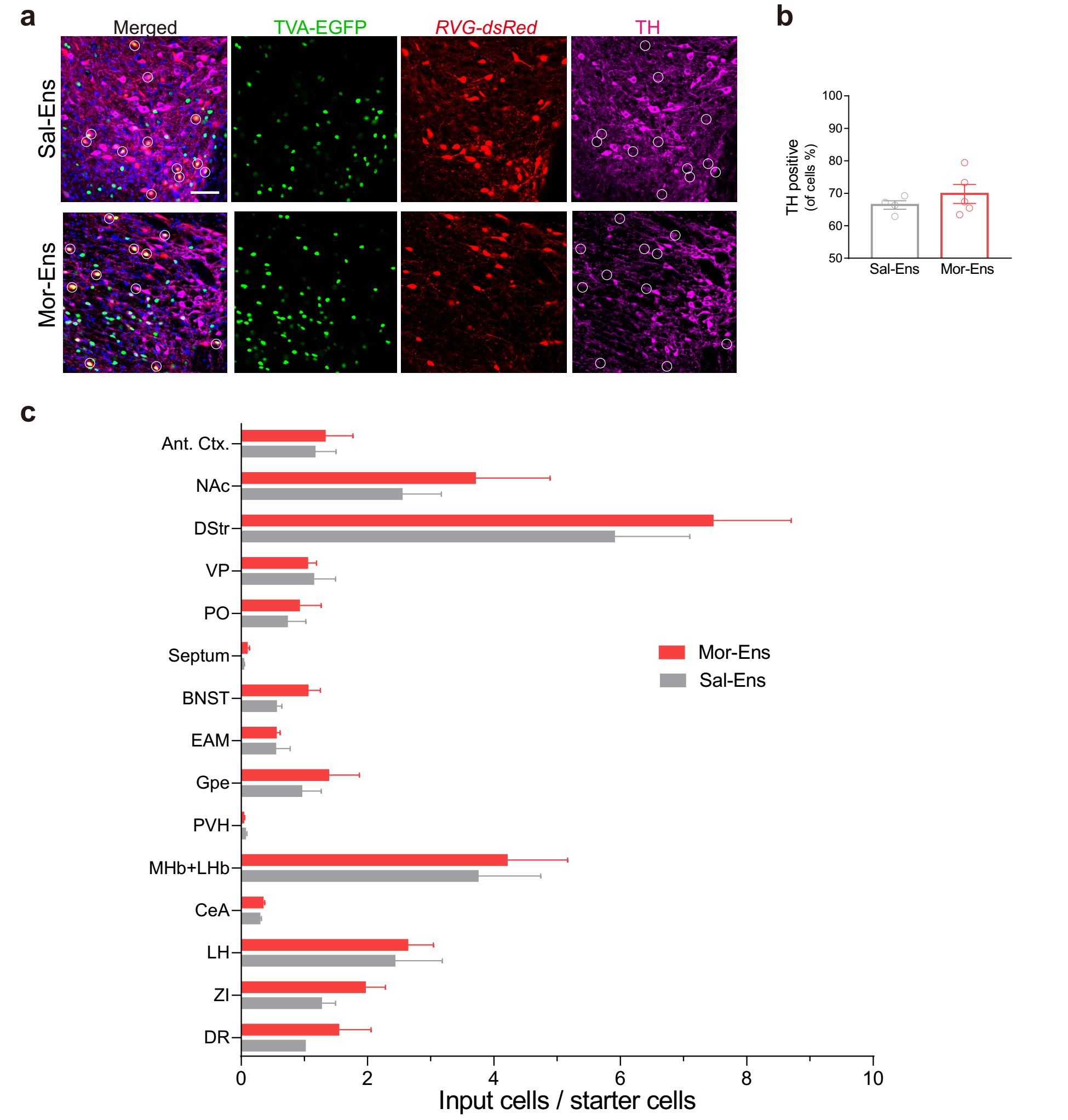


**Figure S9. Identification of the TH^+^ starters and quantification of the inputs on Sal- and Mor-Ens. Related to Figure 4.**

**a-c** Analysis of rabies labeled inputs of the Mor-Ens and Sal-Ens in the VTA. **a** Representative images of TH staining in the VTA. Starter cells co-expressing TVA-GFP (green) and RVG-dsRed (red) in the VTA. Magenta: TH; Blue: DAPI. Scale bar, 50 μm. **b** Quantification of the TH^+^ components in Mor-Ens and Sal-Ens. n = 223 cells from 8 slice of 4 mice in Sal-Ens group; n = 255 cells from 13 slices of 5 mice in Mor-Ens group. Unpaired t test, *t* = 0.9717, *df* = 7, *P* = 0.3636. **c** Brain-wide quantification of inputs (dsRed^+^ cells) to Sal-Ens or Mor-Ens. Data are presented as the input/starter ratio in each group. Number of starter cells, Sal-Ens: 358.4 ± 79.12, n = 5 mice; Mor-Ens: 293 ± 31.28, n = 6 mice. Unpaired t test, *t* = 0.8242 *df* = 9, *P* = 0.4311. Ant.Ctx., anterior cortex; BNST, bed nucleus of the stria terminalis; CeA, central amygdala; DR, dorsal raphe; DStr, dorsal striatum; EAM, extended amygdala; Gpe, globus pallidus; LH, lateral hypothalamus; LHb, lateral habenula; MHb, medial habenula; NAc, nucleus accumbens; PO, preoptic nucleus; PVH, paraventricular hypothalamus; VP, ventral pallidum; ZI, zona incerta. Unpaired t test or Mann-Whitney U test (non-normalized data). Data are presented as mean ± SEM.


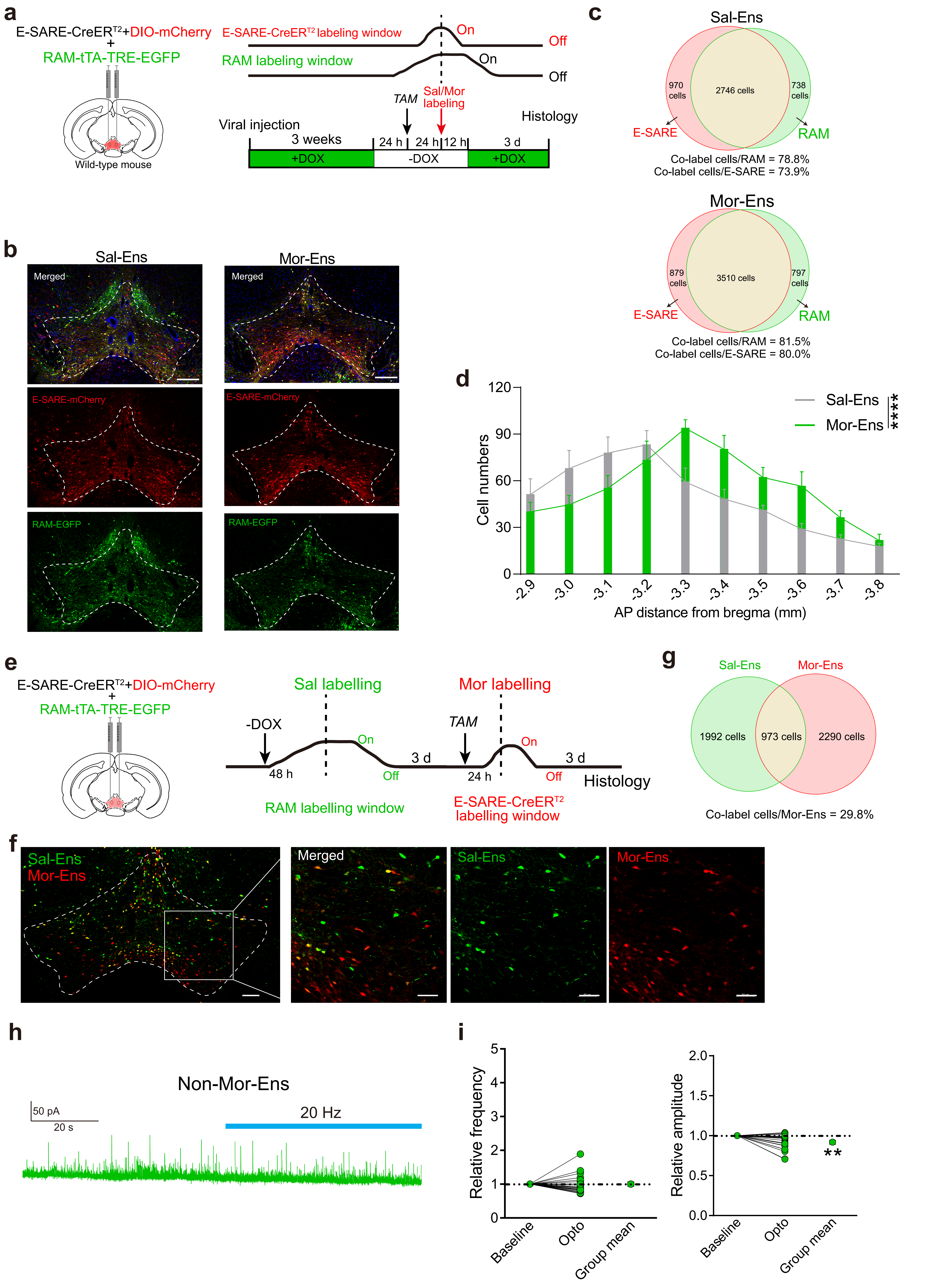


**Figure S10. Identification and quantification of Mor-Ens and Sal-Ens captured by E-SARE and RAM systems. Related to Figure 4.**

**a** Strategy for double systems labeling of Mor-Ens and Sal-Ens. **b** Representative images of the VTA from the mice infected with *DIO-mCherry*, *AAV-E-SARE-Cre^ERT2^*, and *AAV****-****RAM-tTA-TRE-EGFP*. Red: mCherry; Green: EGFP; Blue: DAPI. Scale bar, 100 μm. **c** Quantification of the overlapped ensembles by the E-SARE and RAM system. Top: venn diagram illustrating overlap of E-SARE-capturing and RAM-capturing in Sal-Ens. Down: Venn diagram illustrating overlap of E-SASRE- and RAM-capturing Mor-Ens. n = 7 mice per group. **d** Distribution of Mor-Ens and Sal-Ens labelled with *AAV****-****RAM-tTA-TRE-EGFP* in the VTA across the anterior-posterior axis (7 mice per group). Two-way RM ANOVA, F _(9, 108)_ = 8.088, *P* < 0.0001. **e, f** The intersection of Mor-Ens and Sal-Ens. **e** Strategy for double systems labeling of Mor-Ens by *E-SARE-CreERT2/DIO-mCherry* and Sal-Ens by *RAM-tTA-TRE-EGFP* in the same mouse. **f** Representative images of the VTA from the mice infected with both *DIO-mCherry*, *AAV-E-SARE-Cre^ERT2^*, and *AAV****-****RAM-tTA-TRE-EGFP*. Red: mCherry; Green: EGFP; Blue: DAPI. Scale bar, 50 μm. **g** Venn diagram illustrating the overlapping of Mor-Ens and Sal-Ens. n = 5 mice per group. **h** Representative current trace of the VTA neurons that were not recruited by the initial morphine exposure (EGFP^-^, Non-Mor-Ens) following optical stimulation (473 nm, 5 ms, 20 Hz). **i** The relative frequency and amplitude of mIPSC in Non-Mor-Ens in response to optical stimulation. Paired t test; Frequency, *t* = 2.923, *df* = 20, *P* = 0.0084; amplitude, *t* = 0.5531, *df* = 20, *P* = 0.5863. **P* < 0.05, ***P* < 0.01, *****P* < 0.0001. Data are presented as mean ± SEM.


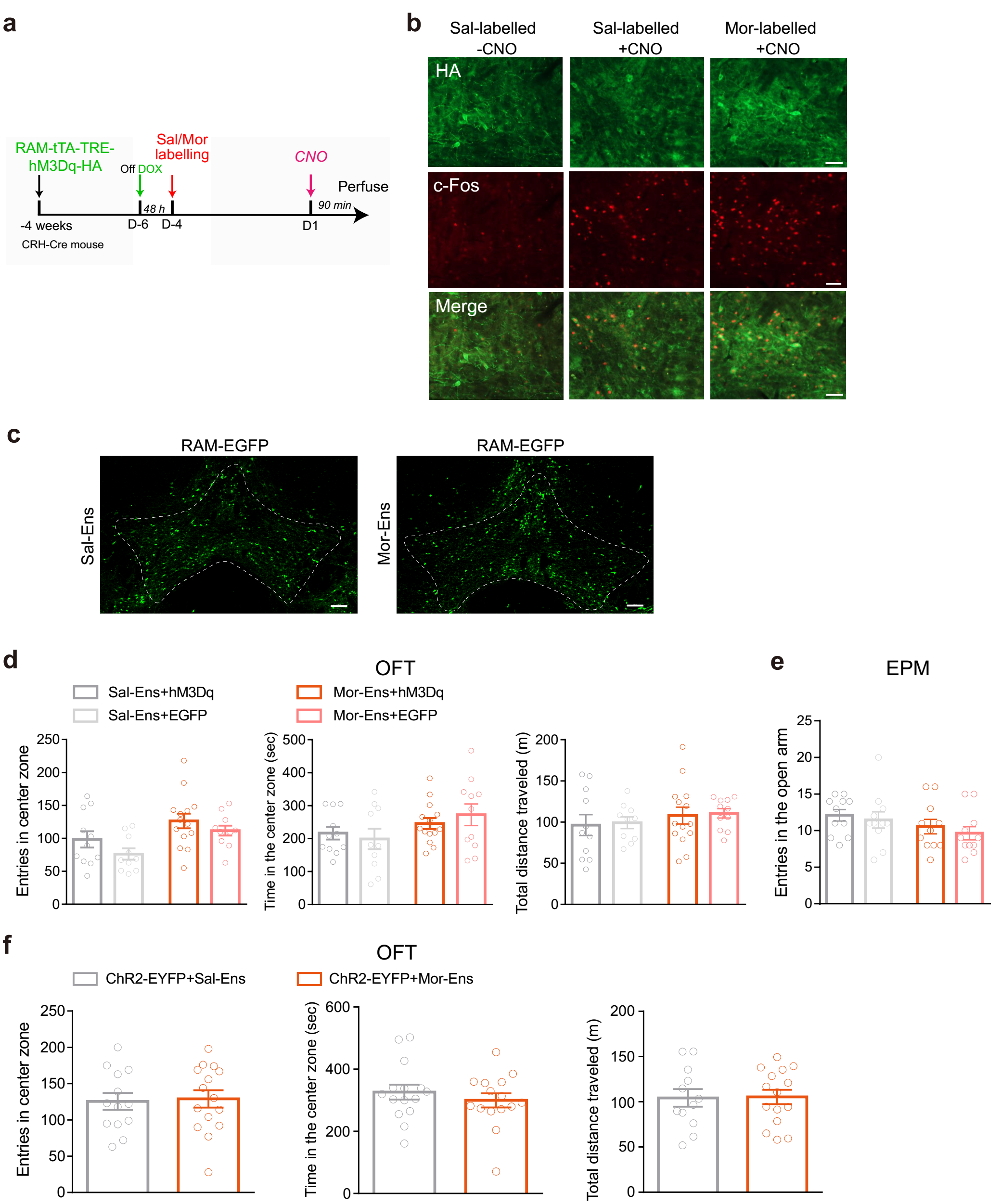


**Figure S11. Activation of Mor-Ens has no effect on the locomotor activity of mice in which the CRH^CeA^**^→^**^VTA^ terminals were optical activated. Related to Figure 5.**

**a** Experimental process of RAM labelling. **b** Representative images of the *hM3Dq-HA* infection and verification of activation in the VTA ensembles by c-Fos staining. Red: c-Fos; Green: HA. Scale bar, 50 μm. **c** Representative image of the *RAM-EGFP* infection in VTA. Green: EGFP. Scale bar, 50 μm. **d, e** The effect of chemogenetic activation of Mor-Ens on the OFT (**d**) and EPM (**e**). Entries in the center zone, Sal-Ens hM3Dq vs Sal-Ens EGFP, *U* = 38.5, *P* = 0.2583; Sal-Ens hM3Dq vs Mor-Ens hM3Dq, *U* = 50.5, *P* = 0.1532; Mor-Ens hM3Dq vs Mor-Ens EGFP, *U* = 63, *P* = 0.4584. Time in the center zone, Sal-Ens hM3Dq vs Sal-Ens EGFP, *U* = 47, *P* = 0.6047; Sal-Ens hM3Dq vs Mor-Ens hM3Dq, *U* = 57, *P* = 0.2915; Mor-Ens hM3Dq vs Mor-Ens EGFP, *U* = 70, *P* = 0.7267. Distance: Sal-Ens hM3Dq vs Sal-Ens EGFP, *U* = 50, *P* = 0.7564; Mor-Ens hM3Dq vs Mor-Ens EGFP, *U* = 69, *P* = 0.8369; Sal-Ens hM3Dq vs Mor-Ens hM3Dq, *U* = 64, *P* = 0.5007. Entries in the open arm, Sal-Ens EGFP vs Sal-Ens hM3Dq, *U* = 48, *P* = 0.4254; Mor-Ens EGFP vs Mor-Ens hM3Dq, *U* = 48.5, *P* = 0.4417; Sal-Ens hM3Dq vs Mor-Ens hM3Dq, *U* = 42, *P* = 0.2315. **f** The effect of optical activation of CRH^CeA→VTA^ terminals and chemogenetic activation of Mor-Ens on the OFT. For entries in the center zone, *P* = 0.8301; the time in the center zone, *P* = 0.6529; distance, *P* = 0.9046. Mann Whitney test. Data are presented as mean ± SEM.


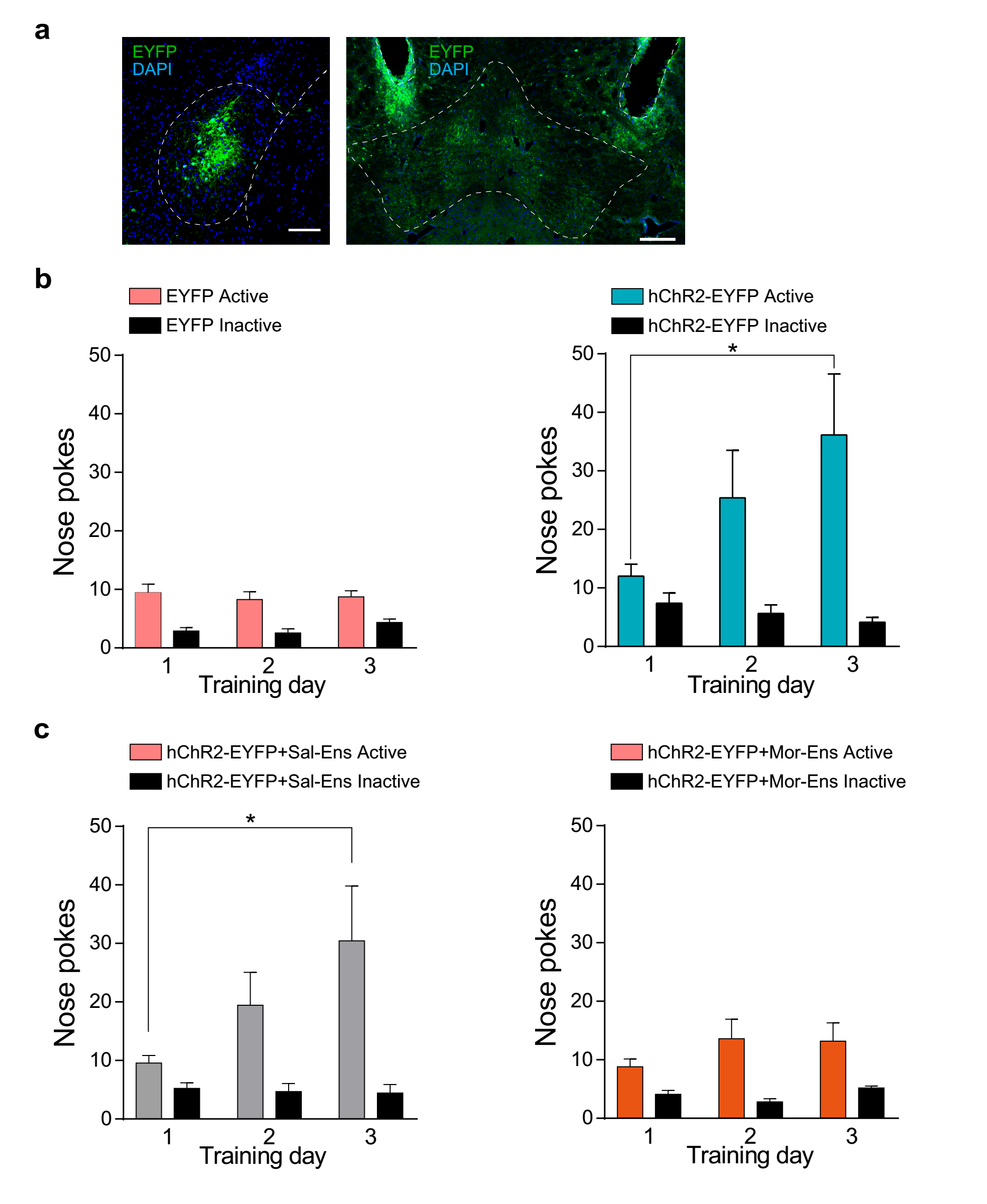


**Figure S12**. **Establishment of nose-poking behavior in the negative reinforcement task. Related to Figure 5.**

**a** Representative image of CRH neurons expressing hChR2 (green) in the CeA and their axon terminals in the VTA. Green: EYFP. Scale bar, 100 μm; **b** The number of active and inactive nose-pokes in EYFP and ChR2-EYFP groups over the first 3-days training sessions. n = 8-11 mice/group. Two-way RM ANOVA, EYFP: F _(2, 40)_ = 1.108, *P* = 0.3400; ChR2-EYFP: F _(2, 28)_ = 1.108, *P* = 0.0142. **c** The number of active and inactive nose-pokes of the mice treated with CNO in Mor-Ens and Sal-Ens groups. n = 9-10 mice/group. Two-way RM ANOVA, hChR2-EYFP+Sal-Ens: F _(2, 32)_ = 4.511, *P* = 0.0188; ChR2-EYFP+Mor-Ens: F _(2, 36)_ = 2.508, *P* = 0.0956. **P* < 0.05. Data are presented as mean ± SEM.


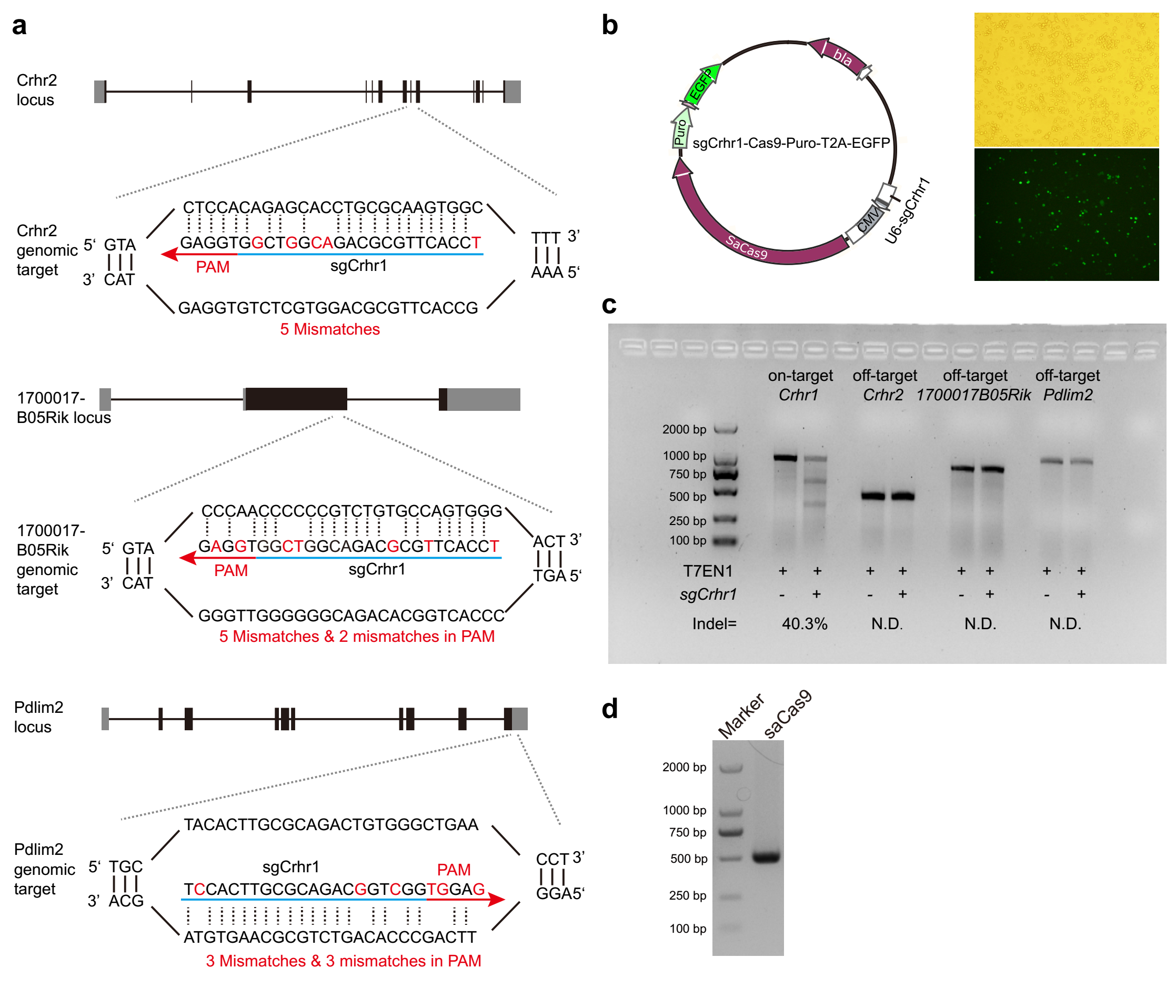


**Figure S13. Analysis of the off-target effects of CRISPR-mediated genome editing, and the expression of *saCas9* in patching neurons by single-cell RT-PCR. Related to Figure 6.**

**a** Predicted off-target site of *sgCrhr1* on *Crhr2*, *1700017B05Rik* and *Pdlim2* exons. **b** Left: Schematic of the vector co-expressing *sgCrhr1* and *saCas9-EGFP*; Right: The expression of EGFP in transfected N2A cells. **c** On-target and off-target indel detection showed that *sgCrhr1* effectively induces mutations in the *Crhr1* (40.3%), but not in the *Crhr2*, *1700017B05Rik* and *Pdlim2* targets. **d** Representative gel images from a single-cell RT-PCR reaction confirming the saCas9 expression in a recorded neuron.


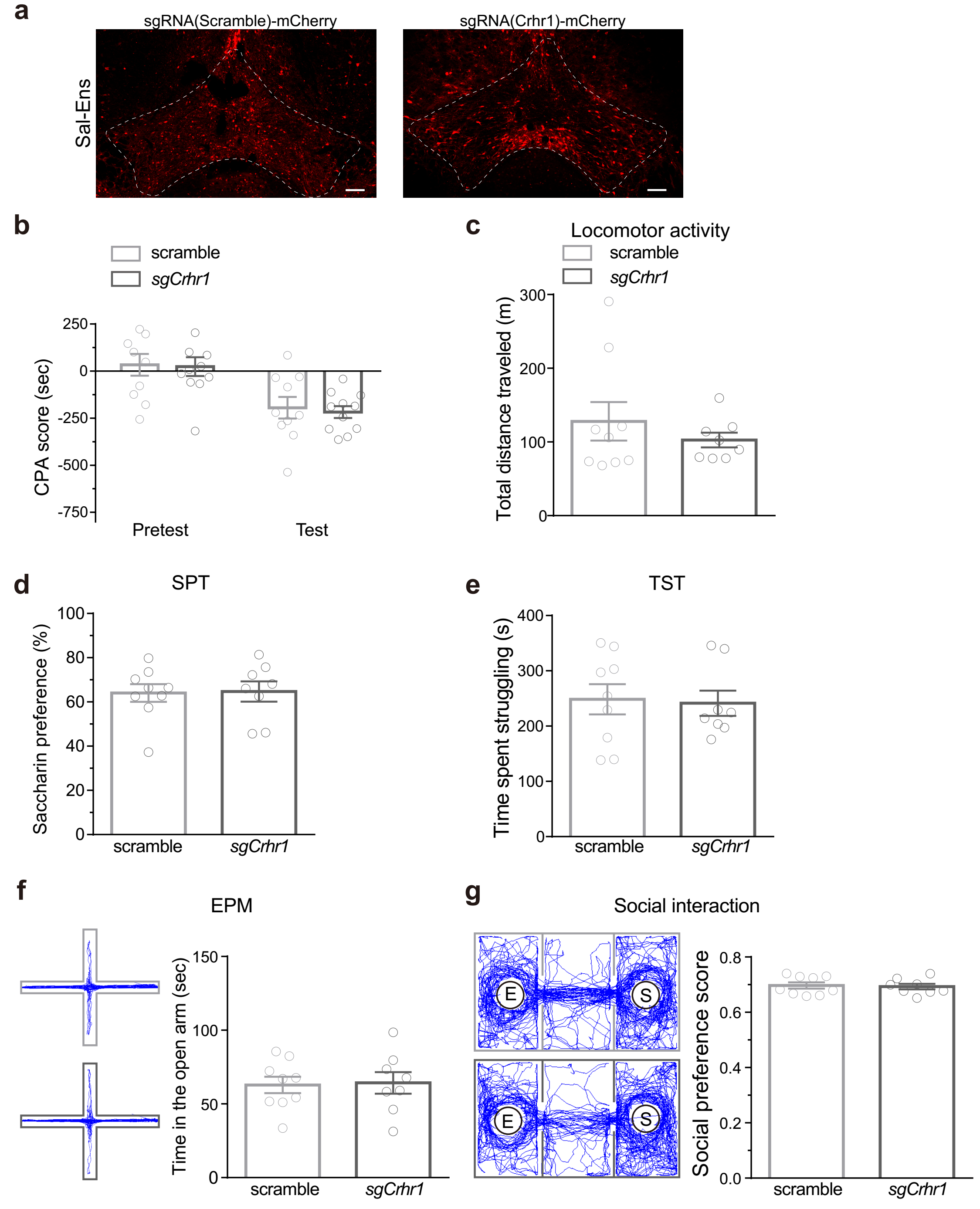


**Figure S14. The effect of CRHR1 deletion in Sal-Ens on the behavioral tests during opiate withdrawal. Related to Figure 6.**

**a** Representative image of *sgRNA(Scramble)-mCherry* and *sgRNA(Crhr1)-mCherry* expressing in VTA. Red, mCherry; Scale bar, 100 μm. **b-g** The effect of CRHR1 deletion in Sal-Ens on withdrawal-induced negative affect. Morphine withdrawal-induced CPA (**b**), OFT (**c**), SPT (**d**), TST (**e**), EPM (**f**), and social interaction (**g**) tests. Two-way RM ANOVA (**b**), F _groups × session_ (1, 19) = 0.0352, *P* = 0.853, scramble *vs* sgCRHR1 within test, *P* > 0.05. (**c**): Unpaired t test, *t* = 0.8594, *df* = 15, *P* = 0.4036. (**d**): *U* = 33, *P* = 0.8148; (**e**): *U* = 33, *P* = 0.8148; (**f**): *U* = 34, *P* = 0.8884; (**g**): *U* = 35, *P* = 0.9626; Mann Whitney test. Data are presented as mean ± SEM.

Supplemental Table1

| REAGENT or RESOURCE | SOURCE | IDENTIFIER |
| --- | --- | --- |
| Antibodies | | |
| Rabbit anti-c-Fos | Santa Cruz | sc-52; RRID:AB_2106783 |
| Mouse anti-c-Fos | ABcam | ab190289; RRID:AB_2737414 |
| Rabbit anti-HA | Sigma-Aldrich | H6908; RRID:AB_260070 |
| Rabbit anti-TH | Millipore | ab152; RRID:AB_390204 |
| Mouse anti-TH | Millipore | MAB318; RRID:AB_2201528 |
| Rabbit anti-CRH | ABcam | ab8901; RRID:AB_306851 |
| In *situ* Probes | | |
| *Cre* | ACDbio | #402551 |
| *Crhr1* | ACDbio | #418011-C3 |
| *mCherry* | ACDbio | #431201-C2 |
| *Crh* | ACDbio | #316091 |
| *dsRed* | ACDbio | #481361-C2 |
| Bacterial and Virus Strains | | |
| *pAAV-E-SARE-CreER^T2^* | Haruhiko Bito (The University of Tokyo) | N/A |
| *pAAV-RAM-TTA-TRE-EGFP* | Addgene | RRID:Addgene_84469 |
| *pAAV-Cre-GFP* | Addgene | RRID:Addgene_68544 |
| *pAAV-hSyn-hM3Dq-mCherry* | Addgene | RRID:Addgene_50474 |
| *pAAV-RAM-TTA-TRE-Flp* | This manuscript | N/A |
| *pAAV-RAM-TTA-TRE-hM3D(Gq)-HA* | This manuscript | N/A |
| *pAAV-RAM-TTA-TRE-Cre* | This manuscript | N/A |
| *pAAV-CMV-sgRNA (Crhr1)-mCherry* | This manuscript | N/A |
| *AAV-EF1α-DIO-hM3Dq-HA* | UNC Vector Core | N/A |
| *AAV-EF1α-DIO-eNpHR3.0-EYFP* | UNC Vector Core | N/A |
| *AAV-EF1α-DIO-hM3Dq-mCherry* | Taitool Bioscience Co., Ltd. | DCJ26 |
| *AAV-EF1α-DIO-hM4Di-mCherry* | Taitool Bioscience Co., Ltd. | DCN28 |
| *AAV-EF1α-DIO-mCherry* | Taitool Bioscience Co., Ltd. | PCF06 |
| *AAV-EF1α-DIO-EGFP* | Taitool Bioscience Co., Ltd. | PBR12 |
| *AAV-Retro-EF1α-DIO-EYFP* | Taitool Bioscience Co., Ltd. | PBI09 |
| *AAV-Retro-EF1α-DIO-mCherry* | Taitool Bioscience Co., Ltd. | N/A |
| *AAV-Retro-EF1α-tdTomato* | Taitool Bioscience Co., Ltd. | N/A |
| *AAV-CAG-fDIO-saCas9* | BrainVTA Co., Ltd | N/A |
| *AAV-CAG-DIO-saCas9* | BrainVTA Co., Ltd | N/A |
| *AAV-EF1α-DIO-hChR2(H134R)-EYFP* | Taitool Bioscience Co., Ltd. | PCD25 |
| *AAV-EF1α-DIO-hChR2(H134R)-mCherry* | Taitool Bioscience Co., Ltd. | PCC16 |
| *CTB-488* | BrainVTA Co., Ltd | CTB-210603 |
| *RV-EnvA-dsRed* | BrainVTA Co., Ltd | 20200928 |
| *AAV-DIO-H2B-TVA-EGFP* | BrainVTA Co., Ltd | 9-23-K200918 |
| *AAV-DIO-RVG* | BrainVTA Co., Ltd | 9-21-K200716 |
| *AAV-hSyn-DA4.4* | BrainVTA Co., Ltd | 9-2134-K191114 |
| *AAV-CMV-sgRNA(scramble)-mCherry* | BrainVTA Co., Ltd | 9-1340-k200407 |
| Chemicals, Peptides, and Recombinant Proteins | | |
| Saccharin | Sigma-Aldrich | 109185; CAS: 81-07-2 |
| Morphine | Shenyang No.1 Pharmaceutical Co., Ltd | CFDA Approval# H21022436 |
| Clozapine N-oxide (CNO) | Sigma-Aldrich | C0832; CAS: 34233-69-7 |
| Biocytin | Sigma-Aldrich | B4261; CAS: 576-19-2 |
| Tamoxifen | Sigma-Aldrich | T5648; CAS: 10540-29-1 |
| Flupenthixol dihydrochloride | Tocris | Cat# 4057; CAS: 2413-38-9 |
| Bicuculline methiodide | Tocris | Cat# 2503; CAS: 40709-69-1 |
| AP-5 | Tocris | Cat# 0106; CAS: 79055-68-8 |
| CNQX | Tocris | Cat# 0190; CAS: 115066-14-3 |
| antalarmin | Sigma-Aldrich | A8727; CAS: 220953-69-5 |
| antisauvagine-30 | Tocris | Cat# 2071; CAS: 220673-95-0 |
| Critical Commercial Assays | | |
| RNAscope® 2.5 Universal Pretreatment Reagents | ACDbio | ACD: 322380 |
| RNAscope® Multiplex Fluorescent Detection Kit v2 | ACDbio | ACD: 323110 |
| Experimental Models: Organisms/Strains | | |
| Mouse: C57BL/6 | The Slaccsas Lab | N/A |
| Mouse: CRH-IRES-Cre | The Jackson Laboratory | JAX stock # 012704 |
| Software and Algorithms | | |
| Patchmaster | HEKA Elektronik | RRID:SCR_000034 |
| Clampfit 10.3 | Molecular Devices | RRID:SCR_011323 |
| Mini Analysis Program | Synaptosoft | RRID:SCR_002184 |
| MATLAB | MathWorks | RRID:SCR_001622 |
| Activity Monitor software | MED Associates | RRID:SCR_014296 |
| Image-Pro Plus 6.0 | Media Cybernetics, Inc | RRID:SCR_016879 |
| SPSS | IBM | RRID:SCR_002865 |
| Prism | Graphpad | RRID:SCR_002798 |
| Etho Vision XT | Leesburg | RRID:SCR_000441 |
